# Promoting Effects of Urinary Proteins from Stone Formers and Influence of Their Physicochemical Properties on Calcium Oxalate Kidney Stone Formation

**DOI:** 10.34133/csbj.0094

**Published:** 2026-05-14

**Authors:** Suttipong Suttapitugsakul, Paleerath Peerapen, Visith Thongboonkerd

**Affiliations:** Medical Proteomics Unit, Research Department, Faculty of Medicine Siriraj Hospital, Mahidol University, Bangkok 10700, Thailand.

## Abstract

Urinary proteins from stone formers/patients have been hypothesized to promote the formation of kidney stones, but with unclear information. In this study, urinary proteins derived from patients with calcium oxalate (CaOx) kidney stones were fractionated based on their isoelectric points using fast protein liquid chromatography with a GigaCap Q-650M column. Individual protein fractions were then purified and examined by multiple CaOx crystal assays, which simulate the stone-forming events, and identified by tandem mass spectrometry. Their physicochemical properties were then analyzed to find their correlations with the crystal-promoting activities. From all 9 fractions (SFQ1 to SFQ9), almost all of them promoted CaOx crystallization, growth, aggregation, and crystal–cell adhesion, and 10, 12, 71, 71, 60, 55, 25, 38, and 6 were identified from SFQ1 to SFQ9, respectively. Among several abundance-weighted physicochemical parameters, molecular weight, instability index, amino acid composition, and secondary structure positively correlated with abundance-weighted crystal aggregation-promoting activity and crystal adhesion-promoting activity. The findings elucidated the roles of urinary proteins from stone formers in kidney stone promotion, contributing to a better understanding of disease mechanisms.

## Graphical Abstract

**Figure:**
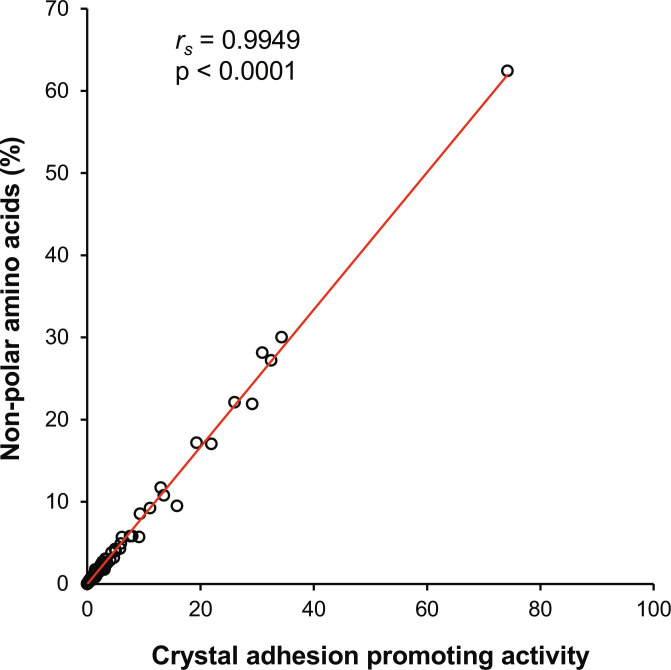

## Introduction

Kidney stone disease (KSD) impacts approximately 11% of the population in the United States, with an annual incidence rate of 2.1% and a recurrence rate exceeding 40% within 4 years [1,2]. The disease occurs when the urine becomes supersaturated with etiologic minerals, especially calcium and oxalate, along with other ions, metabolites, and macromolecules, such as proteins and glycosaminoglycans, all of which make up the stone matrix [3,4]. Most kidney stones consist of calcium oxalate (CaOx) as the predominant crystalline form [5]. KSD patients or stone formers may pass the stones naturally via the urinary passage, while treatment with lithotripsy, ureteroscopy, ureterorenoscopy, or percutaneous nephrolithotomy may be needed [6]. Because of its high prevalence, incidence and recurrence rates with clinical and economic burdens (estimated at billions of US dollars annually), KSD is one of the major healthcare concerns [7,8].

Substantial roles of proteins in the development of KSD have been well documented with evidence for the contributions of their physicochemical properties [3,9–13]. These properties can cause promotion or inhibition of crystallization, growth, aggregation, and crystal–cell adhesion, leading to the formation of kidney stones and the development of KSD [3,9–13]. Certain proteins have calcium- or oxalate-binding sites, acting as the binding agents that facilitate crystallization, crystal growth, and aggregation and mediate interactions with other proteins on the cell membranes, causing crystal–cell adhesion [3,14–17]. Conversely, some proteins with negative/positive charges can attract/repel calcium or oxalate ions or form polycation/polyanion aggregates to either promote or inhibit crystal activities [15,18,19]. A prominent example is Tamm–Horsfall protein (THP), which represents the most abundant protein in human urine. THP has been demonstrated to suppress CaOx aggregation under conditions of elevated pH and reduced ionic strength [20]. However, as pH decreases and ionic strength increases, THP becomes more viscous and promotes crystal aggregation [20]. THP can undergo glycosylation with negatively charged sialic acid-containing N-glycans, and THP sialylation is associated with crystal aggregation and stone formation [21,22]. Other physicochemical properties of proteins, such as hydrophobicity, stability, amino acid composition, and secondary structure, have also been explored, but with scattered information [23–25].

Herein, urine samples were collected from patients with CaOx kidney stones. Urinary proteins were purified and fractionated based on their isoelectric points using fast protein liquid chromatography (FPLC) with a GigaCap Q-650M column. Individual protein fractions underwent multiple CaOx crystal assays to determine their effects on CaOx crystal formation, growth, aggregation, and crystal–cell adhesion. These urinary protein fractions were also analyzed by tandem mass spectrometry (MS/MS) for protein identification, allowing for the determination of their physicochemical properties, such as general parameters, amino acid composition, calcium/oxalate binding, stability/instability, hydrophobicity/hydrophilicity, and secondary structure. These physicochemical properties were then analyzed to find their correlations with the crystal-promoting activities of individual protein fractions with respect to their relative abundance determined by MS/MS analyses.

## Materials and Methods

### Ethics, urine collection, and initial sample treatment

The research design and clinical sample handling were reviewed and approved by the Siriraj Institutional Review Board with the approval number Si415/2023. The signed informed consent forms were obtained from all 30 participants (aged 30 to 74 years). Their random midstream urine samples were collected and centrifuged at 500 *g* for 10 min. Pellets were discarded, whereas 100 ml of the clear supernatants from individual subjects were pooled (to obtain a sufficient amount of proteins for all crystal assays and MS/MS analyses), desalted by dialysis against deionized water, dried by lyophilization, and then resuspended in a buffer containing 10 mM tris-HCl and 50 mM NaCl (pH 7.3).

### Fractionation by diethylaminoethyl anion exchange followed by FPLC based on differential isoelectric points

Urinary protein fractionation was performed by diethylaminoethyl anion exchange followed by FPLC using a GigaCap Q-650M column (Tosoh Bioscience GmbH) as described with greater detail in [26] and Supplementary Methods.

### Measurements of CaOx crystal-promoting activities of SFQ1-SFQ9 urinary protein fractions

Four different CaOx crystal assays were performed in this study, including (a) crystallization, (b) crystal growth, (c) crystal aggregation, and (d) crystal–cell adhesion assays, using an equal volume (4 μl) of 1 μg/μl proteins from each fraction in the crystal buffer, 1 μg/μl lysozyme (Sigma-Aldrich) in the crystal buffer (negative control), or the crystal buffer without any protein (blank control). See details of these assays in [23,27,28] and Supplementary Methods. Also, degrees of CaOx crystal-promoting activities of individual protein fractions were calculated as described with greater detail in Supplementary Methods.

### MS/MS identification of urinary proteins in individual protein fractions

See details in [23,27,28] and Supplementary Methods.

### Defining physicochemical properties of urinary proteins in individual fractions

Various tools were employed to define the physicochemical properties of urinary proteins found in each fraction. Calcium- and oxalate-binding properties are essential features of proteins for their interactions with calcium and oxalate, respectively, in free form and on the crystal surfaces [13,14,29–31]. Calcium-binding domains in individual proteins were identified via UniProt (https://www.uniprot.org*/*) tool, whereas oxalate-binding motifs were identified via the OxaBIND (https://stonemod.org/oxabind.php) tool. Other protein properties, such as molecular weight (MW), isoelectric point, stability, hydropathy (Grand Average of Hydropathicity [GRAVY] score) and residual components, were analyzed via the ProtParam (https://web.expasy.org/protparam/) tool. In addition, their secondary structures were investigated via the SOPMA (https://npsa-prabi.ibcp.fr/cgi-bin/npsa_automat.pl?page=/NPSA/npsa_sopma.html) tool.

### Statistics and correlation analyses

Multiple comparisons were performed using analysis of variance (ANOVA) with Tukey’s post hoc test for normally distributed data; otherwise, Kruskal–Wallis tests were done. Statistical significance was defined as a *P* value of < 0.05. Relative abundance of each identified protein was derived from the MaxQuant data via MS/MS analyses and then used for the determination of abundance-weighted crystal-promoting activities and abundance-weighted physicochemical properties (see details in Supplementary Methods). Correlations between the abundance-weighted crystal-promoting activities and individual abundance-weighted physicochemical properties were assessed by Spearman’s rank correlation analysis. Multiple testing correction was performed using the Benjamini–Hochberg procedure. In addition to an adjusted *P* value of < 0.05, the significant correlation was defined when Spearman’s rank correlation coefficient (*r_s_*) > 0.8 or < −0.8 [32].

## Results

### Crystal-promoting activities of urinary proteins from stone formers fractionated based on isoelectric points

Urine samples were collected from 30 individuals diagnosed with CaOx KSD. Because of the enormous amount of proteins required for all experiments, while urinary protein from each individual was insufficient to do so, all the clarified urine samples were pooled, purified, and fractionated into multiple fractions using FPLC with the GigaCap Q-650M column, which separated proteins based on their decreasing isoelectric points. The fractionated proteins were then again purified and used in various CaOx crystal assays to evaluate their crystal-promoting abilities, whereas the crystal buffer without protein served as the blank control, and lysozyme served as the negative control.

**Fig. 1.**
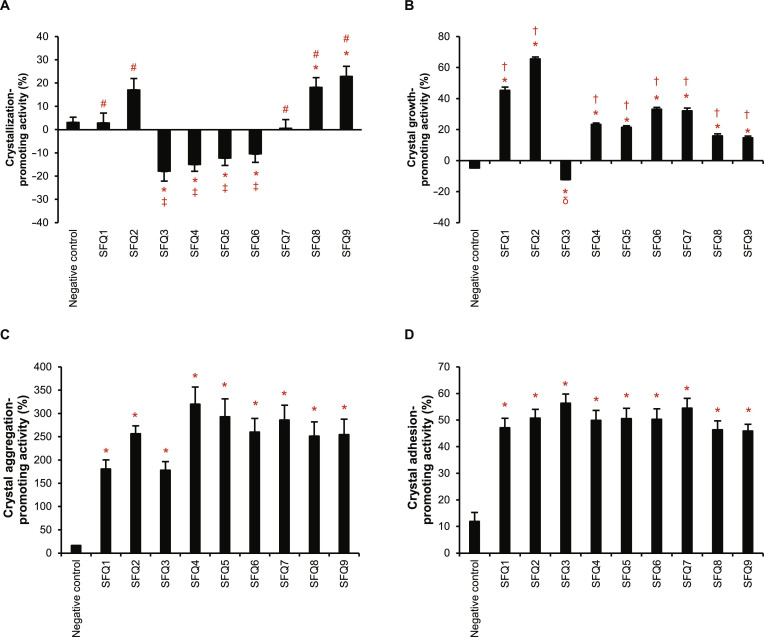
Crystal-promoting activities of urinary proteins from individual fractions. After fractionation, all the protein fractions (SFQ1 to SFQ9) were subjected to crystal assays and determination of their calcium oxalate (CaOx) crystal-promoting activities (see Supplementary Methods). (A) Crystallization-promoting activity. (B) Crystal growth-promoting activity. (C) Crystal aggregation-promoting activity. (D) Crystal adhesion-promoting activity. An error bar represents standard error of the mean (SEM) of the data derived from 3 independent experiments. * = *P* < 0.05 versus negative control, # = *P* < 0.05 versus fraction(s) with crystallization-inhibitory activity, ‡ = *P* < 0.05 versus fraction(s) with crystallization-promoting activity, † = *P* < 0.05 versus fraction(s) with crystal growth-inhibitory activity, and δ = *P* < 0.05 versus fraction(s) with crystal growth-promoting activity.

From a total of 9 urinary protein fractions (SFQ1 to SFQ9), the crystallization assay demonstrated that 5 of them (SFQ1 and SFQ2 and SFQ7 to SFQ9) promoted CaOx crystallization, but only SFQ8 and SFQ9 showed significant difference as compared with the negative control (Fig. 1A). By contrast, fractions SFQ3 to SFQ6 inhibited crystallization (Fig. 1A). Notably, proteins in fractions SFQ1 and SFQ2 and SFQ7 to SFQ9 promoted CaOx crystallization at various degrees, with their averages ranging from 0.6% in SFQ7 to 22.9% in SFQ9, whereas fractions SFQ3 to SFQ6 decreased CaOx crystallization by averages of 10.5% to 18.0%. In the crystal growth assay, all protein fractions, except SFQ3, significantly promoted crystal growth compared with the negative control (Fig. 1B). Proteins in SFQ2 exhibited the greatest promoting effect on crystal growth with an average of 65.5%, whereas other fractions promoted crystal growth from 14.7% to 45.3% on average (Fig. 1B). Proteins in SFQ3 inhibited both crystallization and crystal growth, while SFQ4 to SFQ6 inhibited crystallization but promoted crystal growth (Fig. 1A and B). The crystal aggregation assay revealed that all urinary protein fractions markedly enhanced crystal aggregation compared with the negative control, with their average promoting activities ranging from 178.0% to 320.0% (Fig. 1C). Despite inhibiting CaOx crystallization and growth, proteins in SFQ3 promoted crystal aggregation at an average of 178.0%. Similarly, all the protein fractions markedly enhanced crystal–cell adhesion compared with the negative control, with their average promoting activities ranging from 45.9% to 56.4% (Fig. 1D).

### Analysis of urinary protein physicochemical properties: Calcium- and oxalate-binding potential

MS/MS analyses of urinary proteins following FPLC fractionation identified 10, 12, 71, 71, 60, 55, 25, 38, and 6 from SFQ1 to SFQ9, respectively (Table S1). Among all fractions, SFQ9 and SFQ1 had the smallest numbers of proteins being identified (6 and 10, respectively), while SFQ3 and SFQ4 had the greatest number of proteins being identified (71 in both). Some proteins were found in only 1 fraction (e.g., Tenascin-X in SFQ3), while others were found in more than 1 fraction (e.g., uromodulin and protein AMBP).

**Fig. 2.**
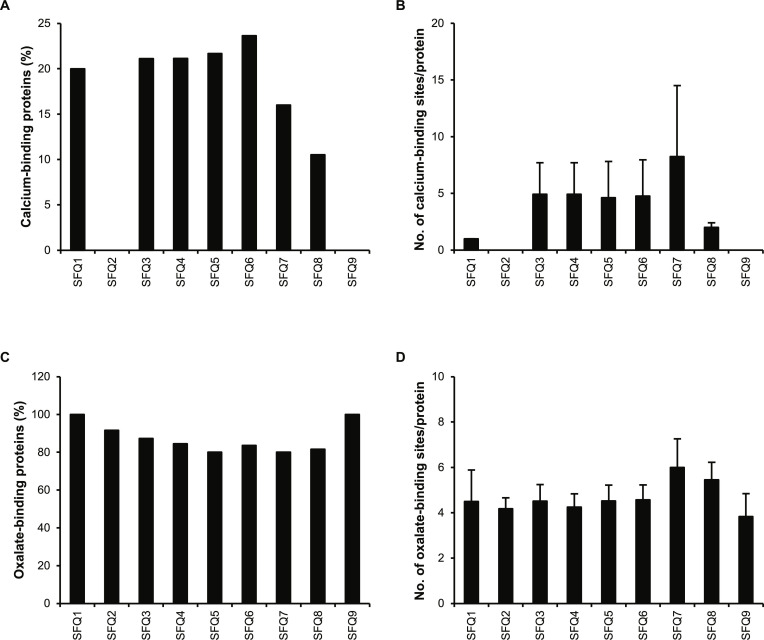
Analysis of calcium- and oxalate-binding sites in urinary proteins identified from individual fractions. After tandem mass spectrometry (MS/MS) protein identification, the calcium- and oxalate-binding potential of proteins identified in individual fractions was analyzed. (A) Proportion of calcium-binding proteins. (B) Number of calcium-binding sites per protein. (C) Proportion oxalate-binding proteins. (D) Number of oxalate-binding sites per protein. An error bar represents standard error of the mean (SEM) of the data derived from all the proteins within each fraction.

Following the identification of proteins by MS/MS, several physicochemical properties of the proteins were analyzed to identify potential factors that might influence or be linked to the crystal-promoting activities. The ion-binding properties, particularly calcium- and oxalate-binding, were first investigated. Of all fractions, 7 contained calcium-binding proteins, ranging from 10.5% in SFQ8 to 23.6% in SFQ6 (Fig. 2A). However, SFQ2 and SFQ9 did not contain any proteins with a calcium-binding domain, possibly due to the small number of proteins being identified in these 2 fractions (Table S1). For the other 7 fractions, the calcium-binding proteins had 1.0 to 8.3 calcium-binding sites per protein on average in each fraction, with fibrillin-1 having the greatest number (43) of calcium-binding sites/protein (Fig. 2B). For oxalate binding, all of the protein fractions contained oxalate-binding proteins. Interestingly, at least 80% (up to 100%) of proteins identified in all fractions had oxalate-binding motifs (Fig. 2C). Notably, while the proteins from SFQ2 and SFQ9 did not have any calcium-binding sites, over 90% of the proteins in these 2 fractions had oxalate-binding motifs. On average, these proteins in individual protein fractions contained 3.8 to 6.0 oxalate-binding motifs per protein (Fig. 2D).

### Analysis of urinary protein physicochemical properties: MW, stability, and hydropathicity

**Fig. 3.**
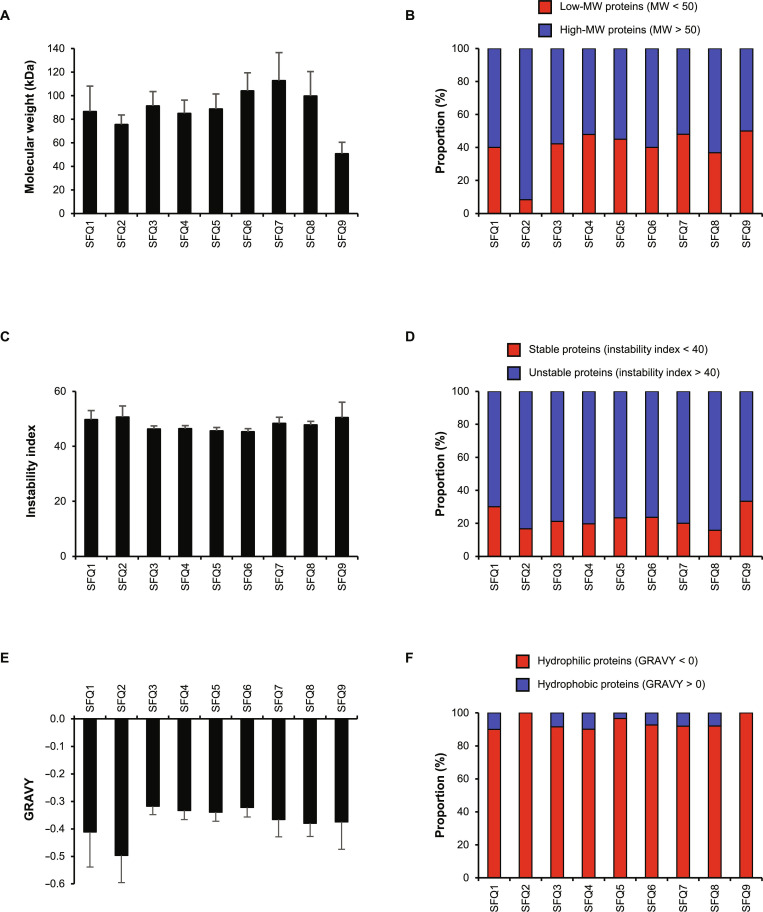
Molecular weight (MW), stability, and hydropathicity of urinary proteins identified from individual fractions. After tandem mass spectrometry (MS/MS) protein identification, various physicochemical properties of proteins identified in individual fractions were analyzed. (A) MW. (B) Distribution of low-MW (<50 kDa) and high-MW (>50 kDa) proteins. (C) Instability index. (D) Distribution of stable (instability index < 40) and unstable (instability index > 40) proteins. (E) Hydropathicity based on GRAVY score. (F) Distribution of hydrophilic (GRAVY < 0) and hydrophobic (GRAVY > 0) proteins. An error bar represents standard error of the mean (SEM) of the data derived from all the proteins within each fraction.

The urine from stone formers contained proteins spanning a broad spectrum of MW, averaging 88.3 kDa across all the fractions (Fig. 3A). SFQ7 had proteins with the highest average MW of 112.8 kDa, while SFQ9 had proteins with the lowest average MW of 50.7 kDa. The largest protein identified was fibrous sheath-interacting protein 2 from SFQ8, with a MW of 780.6 kDa. On the other hand, the smallest protein identified was mucin-like protein 1 from SFQ6, with a MW of 9.0 kDa. All fractions contained a slightly greater proportion of high-MW proteins (MW > 50 kDa) (averaging 60.2% across all fractions) compared with low-MW proteins (MW < 50 kDa) (averaging 39.8% across all fractions). Interestingly, 91.7% of proteins in SFQ2 were high-MW proteins (Fig. 3B).

On average, protein instability index was 47.8 in all fractions (Fig. 3C). Proportionally, most proteins (77.4% on average) in all fractions were unstable with the instability index > 40 (Fig. 3D). SFQ8 contained the greatest proportion of the unstable proteins (84.2%), whereas SFQ9 contained the lowest proportion of the unstable proteins (66.7%) (Fig. 3D). The average of the GRAVY score of proteins in all fractions was −0.37, indicating that they were mainly hydrophilic (Fig. 3E). SFQ2 had the lowest GRAVY score (−0.50 on average), while SFQ3 had the highest GRAVY score (−0.32 on average). In fact, over 90% of the proteins in individual fractions were hydrophilic (GRAVY score < 0) (Fig. 3F). Notably, all proteins in SFQ2 and SFQ9 were predicted to be hydrophilic, as they all had a GRAVY score lower than 0 (Fig. 3F).

### Analysis of urinary protein physicochemical properties: Amino acid composition

**Fig. 4.**
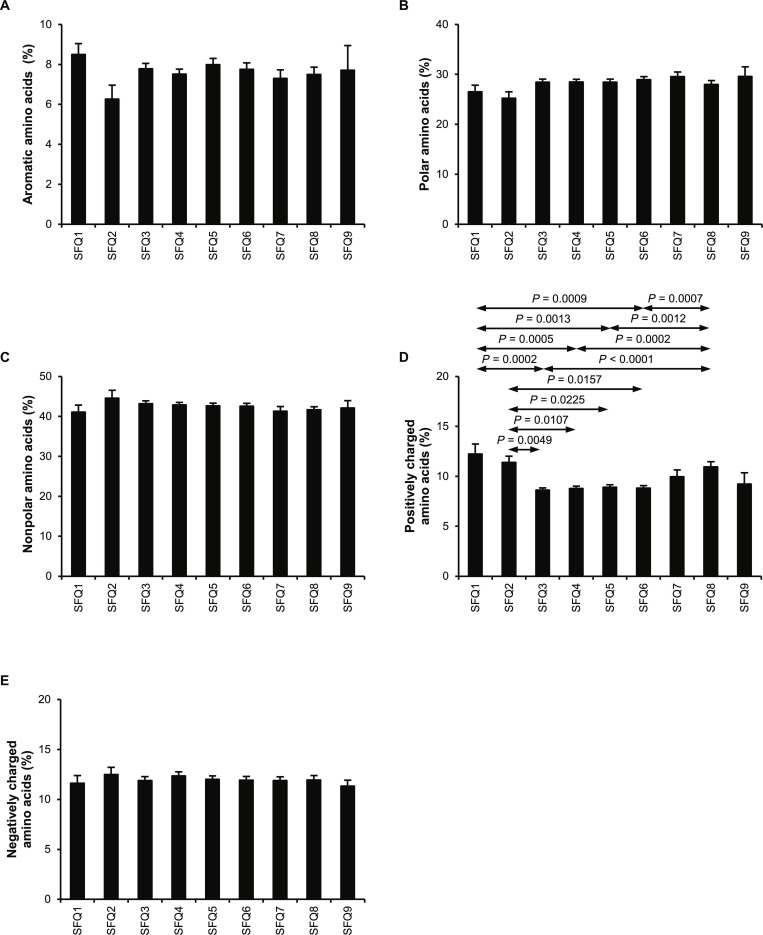
Amino acid composition of urinary proteins identified from individual fractions. After tandem mass spectrometry (MS/MS) protein identification, the amino acid composition of proteins identified in individual fractions was analyzed. (A) Proportion of aromatic amino acids. (B) Proportion of polar amino acids. (C) Proportion of nonpolar amino acids. (D) Proportion of positively charged amino acids. (E) Proportion of negatively charged amino acids. An error bar represents standard error of the mean (SEM) of the data derived from all the proteins within each fraction.

Characteristics of amino acid composition were analyzed. On average, less than 9% of the proteins contained amino acids with aromatic side chains (Fig. 4A). SFQ2 had the lowest average percentage of aromatic amino acids at 6.3%, while SFQ1 had the highest average percentage at 8.5%. Among the identified proteins, beta-hexosaminidase subunit alpha identified from SFQ5 had the highest percentage of aromatic amino acids at 14.4%. The identified urinary proteins contained an average of 25.3% to 29.6% of amino acids with polar side chains (Fig. 4B), while 41.1% to 44.6% on average were amino acids with nonpolar side chains (Fig. 4C). For the charged amino acids, the proteins contained an average of 8.6% to 12.2% of positively charged amino acids (Fig. 4D), while 11.4% to 12.5% on average of amino acids were negatively charged (Fig. 4E). Notably, while proportions of negatively charged amino acids did not show any statistically significant differences across the urine fractions (Fig. 4E), the proportions of positively charged amino acids exhibited statistically significant differences among multiple fractions (Fig. 4D).

### Analysis of urinary protein physicochemical properties: Secondary structure

**Fig. 5.**
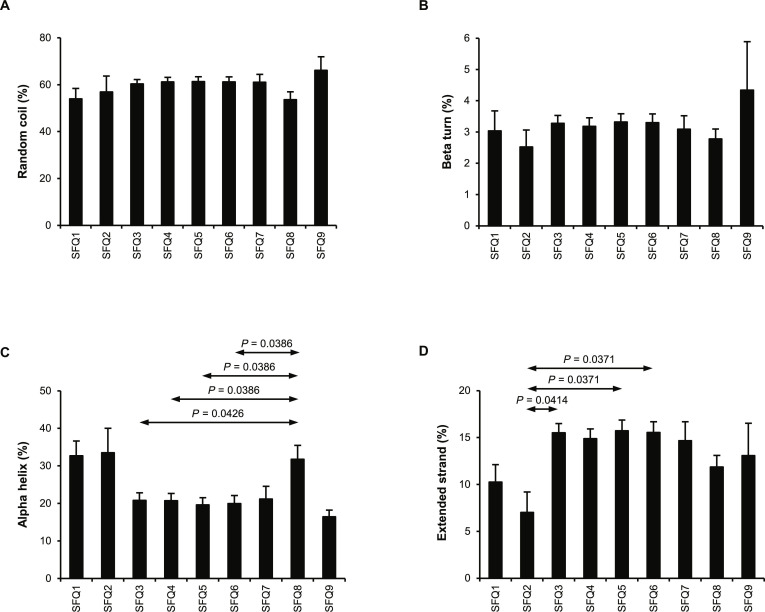
Secondary structure of urinary proteins identified from individual fractions. After tandem mass spectrometry (MS/MS) protein identification, the secondary structure of proteins identified in individual fractions was analyzed. (A) Proportion of random coils. (B) Proportion of beta turns. (C) Proportion of alpha helices. (D) Proportion of extended strands. An error bar represents standard error of the mean (SEM) of the data derived from all the proteins within each fraction.

The secondary structure of proteins was also analyzed through their amino acid sequences. Most protein sequences contained random coils, with average percentages ranging from 53.6% to 66.2% across the fractions (Fig. 5A). By contrast, beta turns occupied only a tiny portion, averaging from 2.5% to 4.3% (Fig. 5B). Alpha helices were found with an average of 16.4% to 33.5%, particularly in SFQ8, in which the percentage of alpha helices was statistically different from (higher than) SFQ3 to SFQ6 (Fig. 5C). The rest were extended strands, averaging from 7.0% to 15.7%, with SFQ2 showing a statistically different (lower) percentage of extended strands compared with SFQ3, SFQ5, and SFQ6 (Fig. 5D). Overall, SFQ9 contained the highest percentages of random coils with an average of 66.2% (Fig. 5A) and beta turns with an average of 4.3% (Fig. 5B), while having the lowest percentage of alpha helices with an average of 16.4% (Fig. 5C). Meanwhile, SFQ2 had the lowest percentages of beta turns (average 2.5%) (Fig. 5B) and extended strands (average 7.0%) (Fig. 5D). Among the identified proteins, progranulin (GRN) identified in SFQ3 to SFQ6 had the highest percentage of random coils at 99.3%. Eukaryotic translation initiation factor 6 identified in SFQ4 had the highest percentage of beta turns at 9.4%. Centrosomal protein of 162 kDa (CEP162) identified in SFQ8 had the highest percentage of alpha helices at 92.6%. Protocadherin-16 (DCHS1) identified in SFQ7 had the highest percentage of extended strands at 34.5%.

### Correlation analysis of abundance-weighted crystal-promoting activities and abundance-weighted physicochemical properties of urinary proteins from stone formers

**Fig. 6.**
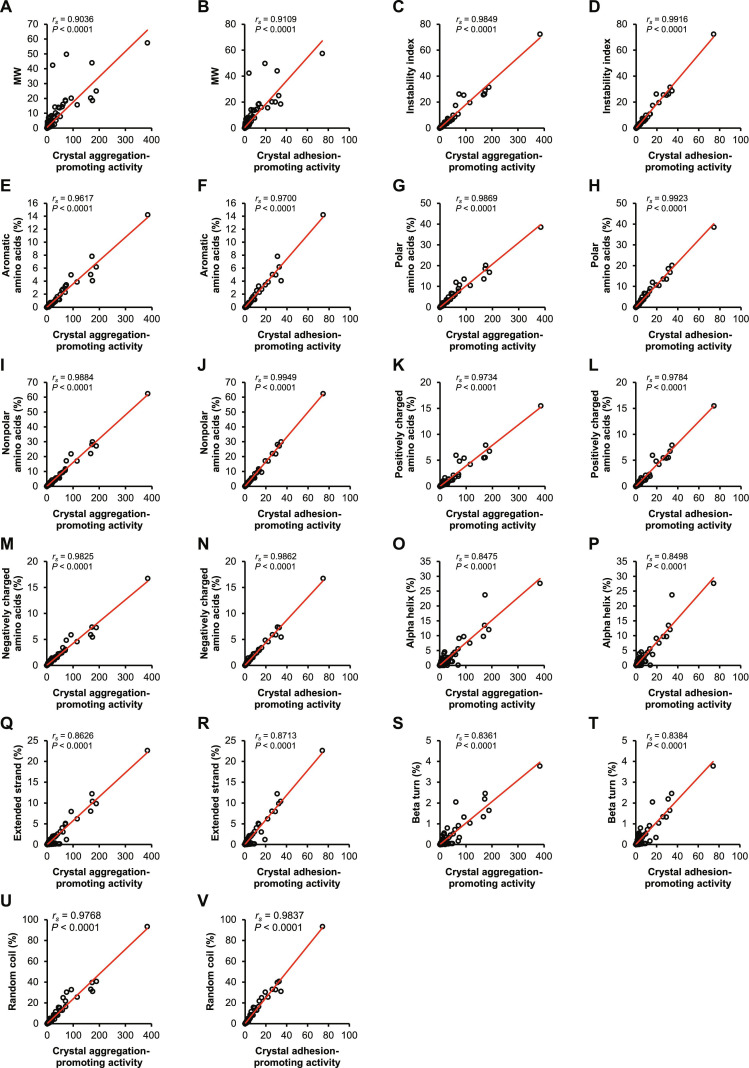
Spearman’s rank correlation analysis between abundance-weighted protein physicochemical properties and abundance-weighted crystal-promoting activities. Correlations between abundance-weighted protein physicochemical properties and abundance-weighted crystal-promoting activities were assessed by Spearman’s rank correlation analysis (see details in Table 1). Only significant correlations (Spearman’s rank correlation coefficient, *r_s_*, > 0.8 or < −0.8 with adjusted *P* value < 0.05) are illustrated here, including abundance-weighted MW (A and B), instability index (C and D), amino acid composition (E to N), and secondary structure (O to V), which positively correlated with abundance-weighted crystal aggregation-promoting activity and crystal adhesion-promoting activity.

Experimental data described above showed 2 aspects of differences found among individual fractions of urinary proteins from stone formers. The crystal assays showed different degrees of CaOx crystal-promoting activities, whereas MS/MS analyses showed some differences in physicochemical properties of urinary proteins in differential fractions. We then attempted to determine correlations between these 2 aspects of differences. As MS/MS analyses also revealed the relative abundance of proteins within each fraction, the crystal-promoting activities and physicochemical properties were normalized based on their relative abundance. Correlations between abundance-weighted crystal-promoting activities and abundance-weighted physicochemical properties of proteins in individual fractions were then examined using Spearman’s rank correlation test (because the data were not normally distributed). After filtering by Spearman’s correlation coefficient (*r_s_*), 22 significant correlations arose (Table 1 and Fig. 6A to V). These included positive correlations between abundance-weighted MW, instability index, amino acid composition (including percentages of aromatic, polar, nonpolar, positively charged, and negatively charged amino acids) and secondary structure (including percentages of alpha helix, extended strand, beta turn, and random coil), and abundance-weighted crystal aggregation-promoting activity and crystal adhesion-promoting activity (Table 1 and Fig. 6A to V).

**Table 1.** Spearman’s rank correlation analysis between abundance-weighted protein physicochemical properties and abundance-weighted crystal-promoting activities. The bold values are accompanied with the superscript “b”, which has been clearly defined at the table footnotes.

Properties		*r_s_* (Adjusted *P* value) ^a^	Crystallization-promoting activity	Crystal growth-promoting activity	Crystal aggregation-promoting activity	Crystal adhesion-promoting activity
Calcium-/Oxalate-binding potential	Calcium-binding proteins (%)	*r_s_*	−0.7983	−0.0252	0.3109	0.2941
		(*P* value) ^a^	(0.0167)	(0.9707)	(0.5456)	(0.5724)
	No. of calcium-binding sites/protein	*r_s_*	−0.1926	0.1290	0.2619	0.2637
		(*P* value) ^a^	(0.0005)	(0.0261)	(<0.0001)	(<0.0001)
	Oxalate-binding proteins (%)	*r_s_*	0.3277	0.1008	−0.5462	−0.3530
		(*P* value) ^a^	(0.5190)	(0.9101)	(0.1978)	(0.4759)
	No. of oxalate-binding sites/protein	*r_s_*	−0.1188	0.5165	0.7421	0.7502
		(*P* value) ^a^	(0.0419)	(<0.0001)	(<0.0001)	(<0.0001)
MW	MW	*r_s_*	−0.2194	0.6174	**0.9036** ^b^	**0.9109** ^b^
		(*P* value) ^a^	(0.0001)	(<0.0001)	(<0.0001)	(<0.0001)
	Low-MW proteins (%)	*r_s_*	−0.1506	−0.4603	0.3682	−0.0669
		(*P* value) ^a^	(0.8200)	(0.3117)	(0.4603)	(0.9274)
	High-MW proteins (%)	*r_s_*	0.1506	0.4603	−0.3682	0.0669
		(*P* value) ^a^	(0.8092)	(0.3066)	(0.4531)	(0.9162)
Stability	Instability index	*r_s_*	−0.2352	0.6690	**0.9849** ^b^	**0.9916** ^b^
		(*P* value) ^a^	(<0.0001)	(<0.0001)	(<0.0001)	(<0.0001)
	Stable proteins (%)	*r_s_*	0.0500	−0.0833	−0.1667	−0.2667
		(*P* value) ^a^	(0.9411)	(0.9143)	(0.8282)	(0.6223)
	Unstable proteins (%)	*r_s_*	−0.0500	0.0833	0.1667	0.2667
		(*P* value) ^a^	(0.9301)	(0.9030)	(0.8167)	(0.6134)
Hydropathicity	GRAVY	*r_s_*	0.0571	−0.5048	−0.6599	−0.6604
		(*P* value) ^a^	(0.4089)	(<0.0001)	(<0.0001)	(<0.0001)
	Hydrophilic proteins (%)	*r_s_*	0.4854	−0.0251	0.1590	−0.0837
		(*P* value) ^a^	(0.2812)	(0.9598)	(0.8231)	(0.9370)
	Hydrophobic	*r_s_*	−0.4854	0.0251	−0.1590	0.0837
	proteins (%)	(*P* value) ^a^	(0.2765)	(0.9489)	(0.8120)	(0.9251)
Amino acid composition	Aromatic amino acids (%)	*r_s_*	−0.2409	0.6534	**0.9617** ^b^	**0.9700** ^b^
		(*P* value) ^a^	(<0.0001)	(<0.0001)	(<0.0001)	(<0.0001)
	Polar amino acids (%)	*r_s_*	−0.2470	0.6690	**0.9869** ^b^	**0.9923** ^b^
		(*P* value) ^a^	(<0.0001)	(<0.0001)	(<0.0001)	(<0.0001)
	Nonpolar amino acids (%)	*r_s_*	−0.2441	0.6695	**0.9884** ^b^	**0.9949** ^b^
		(*P* value) ^a^	(<0.0001)	(<0.0001)	(<0.0001)	(<0.0001)
	Positively charged amino acids (%)	*r_s_*	−0.2040	0.6704	**0.9734** ^b^	**0.9784** ^b^
		(*P* value) ^a^	(0.0002)	(<0.0001)	(<0.0001)	(<0.0001)
	Negatively charged amino acids (%)	*r_s_*	−0.2435	0.6691	**0.9825** ^b^	**0.9862** ^b^
		(*P* value) ^a^	(<0.0001)	(<0.0001)	(<0.0001)	(<0.0001)
Secondary structure	Alpha helix (%)	*r_s_*	−0.1256	0.5989	**0.8475** ^b^	**0.8498** ^b^
		(*P* value) ^a^	(0.0306)	(<0.0001)	(<0.0001)	(<0.0001)
	Extended strand (%)	*r_s_*	−0.2259	0.5816	**0.8626** ^b^	**0.8713** ^b^
		(*P* value) ^a^	(<0.0001)	(<0.0001)	(<0.0001)	(<0.0001)
	Beta turn (%)	*r_s_*	−0.1351	0.5904	**0.8361** ^b^	**0.8384** ^b^
		(*P* value) ^a^	(0.0193)	(<0.0001)	(<0.0001)	(<0.0001)
	Random coil (%)	*r_s_*	−0.2618	0.6568	**0.9768** ^b^	**0.9837** ^b^
		(*P* value) ^a^	(<0.0001)	(<0.0001)	(<0.0001)	(<0.0001)

MW, molecular weight; GRAVY, Grand Average of Hydropathicity

^a^ Adjusted *P* value determined by the Benjamini–Hochberg procedure.

^b^ The significant correlation was defined when the adjusted *P* value < 0.05 AND *r_s_* > 0.8 or < −0.8.

## Discussion

The development of kidney stones can be affected by various factors, including but not limited to genetic predispositions, environmental influences, dietary habits, and medication [33–35]. Frequently, there is a supersaturation of minerals, such as calcium and oxalate, in the urine of patients with KSD. Following a nucleation event that initiates crystallization, the crystals may grow further and then aggregate. With crystal retention through adhesion to epithelial cells along the renal or urinary tract, kidney stones then form [3,36,37]. Another important factor affecting kidney stone formation is the presence of low-MW compounds, e.g., citrate, alongside high-MW compounds, including proteins and glycosaminoglycans [3,38–42]. Throughout crystallization, growth, aggregation, and adhesion processes, proteins may directly interact with the crystals to either promote or inhibit stone formation or indirectly affect the crystals by altering their surrounding environment [43]. Previous works have investigated the roles of proteins in KSD, focusing on the identification of proteins in the urine of KSD patients or individuals without KSD to determine proteins that have the potential to either facilitate or impede the formation of kidney stones, respectively [44,45]. Others have investigated the proteins that constitute the stones, which require an effective method for extracting proteins inside them prior to protein identification [46–49]. However, the presence of urinary proteins inside the stones implicates their promoting effects on kidney stone formation, necessitating the experimental determination of their stone-promoting activities and properties to examine their roles in KSD.

In this study, urine samples were collected from patients diagnosed with KSD to examine the roles of proteins in promoting CaOx stone formation, particularly crystallization, crystal growth, aggregation, and adhesion to renal cells. Initially, proteins were subjected to purification by diethylaminoethyl anion exchange. Previous research has suggested that negatively charged proteins may facilitate kidney stone formation by binding to the positively charged calcium ions present on the crystal surfaces and effectively acting as a glue that binds crystals together to form stones [50,51]. Additionally, other studies have shown that both negatively and positively charged proteins can form aggregates that promote kidney stone formation at certain pH levels, as proteins found in kidney stones generally have isoelectric points <5 or >9 [15,52]. To further analyze urinary proteins and their CaOx crystal-promoting activities, we fractionated them using a GigaCap Q-650M, an anion exchange column that separates the complex protein sample based on differential isoelectric points. It is important to note that anion exchange resins may lead to the depletion or removal of some proteins that could influence kidney stone formation. After fractionation, the protein fractions obtained (SFQ1 to SFQ9) were subjected to CaOx crystal assays and MS/MS analyses.

The data obtained from crystal assays have indicated that urinary protein fractions significantly contribute to various kidney stone formation processes and emphasized the diverse functions of specific protein fractions, which underscore the intricate nature of stone formation processes and the multifaceted roles of urinary proteins. From all 9 protein fractions, SFQ1, SFQ2, SFQ7, SFQ8, and SFQ9 were found to enhance all 4 crystal formation processes (Fig. 1). The crystallization assay demonstrated that only 5 out of the 9 protein fractions had positive crystallization-promoting activity values, indicating that they promoted CaOx crystallization, while the remaining fractions inhibited the process (Fig. 1A). The initiation of crystallization requires supersaturation and the organization of ions into an appropriate lattice structure. Proteins present in the surrounding environment may affect this process by offering ionic patterns on their surfaces that support the development of the initial crystal lattice. This implies that certain urine fractions might be deficient in the proteins required for such a phenomenon [53]. Conversely, some proteins may bind to free calcium or oxalate ions in solution, thereby preventing crystal formation [54]. Despite this, 3 of the crystallization-inhibitory fractions (SFQ4 to SFQ6) showed positive crystal growth-promoting activities, indicating that while they inhibited crystallization, they promoted CaOx crystal growth (Fig. 1B). All protein fractions were observed to facilitate the aggregation of CaOx crystals and the adhesion of crystals to cells (Fig. 1C and D). The most significant impact of these urinary proteins derived from stone formers was observed in the crystal aggregation process, with aggregation increasing by >150% in all the fractions (Fig. 1C). This dramatic increase in crystal aggregation underscores the potential for these proteins to significantly influence kidney stone formation. Lysozyme, which was used as the negative control, showed very low degrees of crystal-modulatory activities compared with all urinary protein fractions (Fig. 1), further supporting that the crystal-promoting properties arose from specific urinary proteins obtained from stone formers, not from the general effects of proteins at the same amount and concentration used in all the crystal assays.

Previous studies have shown that some proteins can form a coating layer around crystals with a thickness of 10 to 20 nm [55,56]. In a crystal growth study using atomic-force microscopy, the principal mechanism for the growth of CaOx crystals involves the progressive incorporation of ions through surface adsorption [57]. Proteins may also increase the crystal surface charges and thus promote the attachment of opposite charges, i.e., free ions, charges on other crystals, and charges on the cell membranes, to the crystals, leading to enhancement of crystal growth, aggregation, and crystal–cell adhesion, respectively [58,59]. However, some proteins may adhere to the crystal surfaces to prevent further crystal growth, while others may attract additional ions to form larger crystals or aggregates [43]. Nonetheless, the roles of some proteins in stone formation are dual, as they can promote stone formation at some steps while inhibiting other steps. It is thus essential to emphasize that effects from all proteins in each fraction were combined and utilized collectively to affect individual crystal assays. For instance, 71 proteins were identified from SFQ3, including vitronectin, thrombomodulin, and immunoglobulins (Table S1), and SFQ3 was found to inhibit both the crystallization and crystal growth processes. Some proteins were identified only in this fraction (e.g., vitamin D-binding protein and tenascin-X) and thus might be responsible for the negative effects on crystallization and crystal growth. Vitamin D-binding protein has a negative correlation with KSD, as the absence of vitamin D-binding protein could result in severe vitamin D deficiency [60], and high vitamin D level is associated with KSD in some patients due to hypercalciuria [61]. Tenascin-X has been proposed as a novel antilithiatic protein in a recent multiplexed proteomics quantification study [62]. The presence of these 2 proteins might be partly responsible for the inhibition of crystallization and crystal growth by SFQ3. Nevertheless, SFQ3, on the other hand, promoted crystal aggregation and crystal–cell adhesion, indicating the predominant influence of other proteins present in the SFQ3 fraction on these 2 processes.

The utilization of MS/MS analyses to identify proteins also revealed their relative abundance, allowing for the determination of abundance-weighted crystal-promoting activities and facilitating the examination of their physicochemical properties by using a wide range of bioinformatics tools. Proteins’ properties that might affect kidney stone formation were studied, including general parameters, amino acid composition, calcium/oxalate binding, stability/instability, hydrophobicity/hydrophilicity, and secondary structure (Table 1). Factors that were determined to affect kidney stone formation included MW, instability index, amino acid composition, and secondary structure, which were found to positively correlate with the abundance-weighted crystal aggregation-promoting activity and crystal adhesion-promoting activity (with *r_s_* > 0.8 and adjusted *P* value < 0.0001) (Table 1).

Analysis of ion-binding potential showed that less than 25% of the proteins in each fraction had calcium-binding domain(s), with 2 fractions (SFQ2 and SFQ9) lacking any calcium-binding proteins (Fig. 2A and B). On the other hand, >80% of the proteins in each fraction contained multiple oxalate-binding motifs (3.8 to 6.0 motifs per protein) (Fig. 2C and D), implying that the general crystal-promoting activities of these protein fractions might be mainly due to the presence of these oxalate-binding motifs. However, neither the abundance-weighted calcium-binding nor oxalate-binding property exhibited any positive or negative correlations with the crystal-promoting activities (Table 1). The presence of calcium- and oxalate-binding sites in these proteins could lead to their binding with calcium and/or oxalate ions present on the crystal surfaces or their free ions present in the solution, affecting the 4 crystal processes differently. The binding of these proteins with the free ions might prevent them from crystallization and growth processes, thereby inhibiting the crystallization and growth activities. On the other hand, their binding with the solid crystal phase might facilitate the recruitment of more ions to enlarge the crystals, their crystal–crystal interactions and crystal interactions with renal cells, thereby promoting crystal growth, aggregation, and adhesion activities. The lack of their positive and negative correlations might be due to the mixture of proteins in each fraction, in which different proteins might interplay, and their crystal modulatory activities might be averaged.

The MW of proteins may also influence the formation of CaOx crystals, as proteins with different sizes and chain lengths may adhere differently to various crystal faces. All protein fractions examined herein exhibited a similar distribution of low-MW (MW < 50 kDa) and high-MW (MW > 50 kDa) proteins, except SFQ2, which contained 8.3% low-MW proteins on average, compared with an average of 39.8% for other fractions (Fig. 3B). The abundance-weighted MW was found to positively correlate with the abundance-weighted crystal aggregation-promoting activity and crystal adhesion-promoting activity (Table 1 and Fig. 6A and B). The higher MW suggested the proteins contain several functional groups or motifs that could facilitate crystal–crystal and crystal–cell interactions. It is also important to acknowledge that proteins can undergo posttranslational modifications, such as glycosylation, which may alter their apparent MW and have been reported to impact kidney stone formation [22]. Nevertheless, these were not analyzed in the present study, as the experimental design would require an enrichment of glycosylated species prior to MS/MS analyses. Herein, we only considered the protein MW without any posttranslational modifications. The size of proteins also increases the diversity of amino acid side chains, thereby increasing the functional groups that can influence kidney stone formation processes.

Similarly, all 5 amino acid composition parameters, including percentages of aromatic, polar, nonpolar, positively charged, and negatively charged amino acids, positively correlated with the abundance-weighted crystal aggregation-promoting activity and crystal adhesion-promoting activity (Table 1 and Fig. 6E to N). Polar and nonpolar amino acids, including the aromatic ones such as phenylalanine, tyrosine, and tryptophan, have been found in the calcium-binding sites of several proteins, especially those categorized as calcium-sensing proteins [63–66]. Negatively charged amino acids, including glutamic acid and aspartic acid, have also been identified in the calcium-binding sites [67,68]. On the other hand, positively charged amino acids such as lysine and arginine are considered a crucial part of the oxalate-binding motifs [54,69,70]. Collectively, the higher MW also increased the chance of the presence of these amino acids, resulting in the greater binding potential of proteins to CaOx crystals, thereby promoting crystal aggregation and crystal–cell adhesion. Interestingly, SFQ2 had the most significant impact on crystal growth, with an average effect of 65.5% compared with other fractions (Fig. 1B), and influenced crystallization by 17.1% on average (Fig. 1A). SFQ2 also had the lowest percentages of aromatic and polar amino acids (Fig. 4A and B), while having the highest percentages of nonpolar and negatively charged amino acids (Fig. 4C and E), which could enhance cation binding. Despite containing mostly large proteins with more amino acid residues, SFQ2 did not contain any calcium-binding proteins (Fig. 2A).

Protein stability and hydropathicity are key factors that determine whether proteins will be denatured or form aggregates in water. Unstable proteins (with an instability index over 40) and hydrophobic proteins (with a GRAVY score above 0) are more likely to aggregate or precipitate [71]. Previous investigations have shown that protein aggregates can serve as the starters for the formation and aggregation of CaOx crystals [36,72]. Herein, most of the urinary proteins identified in this study were hydrophilic, with the GRAVY scores below 0 (Fig. 3E and F). This has been expected since urinary proteins need to stay soluble in the environment of the urine. However, despite being hydrophilic, most of the proteins analyzed were predicted to be unstable, with instability indices over 40. This suggests that while these proteins were soluble, they might still be prone to denaturation and aggregation under certain conditions. Interestingly, the abundance-weighted instability index positively correlated with the abundance-weighted crystal aggregation-promoting activity and crystal adhesion-promoting activity (Table 1 and Fig. 6C and D).

The secondary structure of proteins was lastly examined, revealing that most proteins adopted random coil structure, while only a tiny portion had beta turn structure (Fig. 5A and B). Notably, all 4 forms of the secondary structure positively correlated with the abundance-weighted crystal aggregation-promoting activity and crystal adhesion-promoting activity (Table 1 and Fig. 6O to V). Previous studies have shown that protein sequences adopting various secondary structures, especially extended strands, are associated with ion binding [73–77]. Extended strands (also referred to as beta-strands) are structural elements of beta-sheets. These beta-sheets are stabilized through hydrogen bond interactions between the backbone atoms of adjacent strands. This creates a relatively rigid and planar structure with exposed side chains that can interact with various ions [78,79]. Calcium ions, in particular, have a high affinity for binding to negatively charged or polar side chains, such as those found in aspartic acid and glutamic acid residues, which are often present in extended strands, although not as much as those of isoleucine [80,81]. Similarly, oxalate ions are capable of interacting with positively charged or polar side chains within the extended strands. Furthermore, the rigidity and stability of the extended strands may enhance the structural integrity of proteins upon binding with these ions, potentially affecting the proteins’ functions and roles in stone formation processes, such as the inhibition of crystal growth. Hence, as more extended strands are present, binding with ions may be enhanced, and crystal aggregation and crystal–cell adhesion are also positively affected, as demonstrated in this work.

While the results showed that urinary proteins from stone formers affected CaOx stone formation processes, particularly with positive correlations between their physicochemical properties and the enhancement of crystal aggregation and crystal–cell adhesion phases, some limitations of this work existed. First, the urine samples were collected from 30 patients with CaOx stones and then pooled to obtain a sufficient amount of proteins for all crystal assays and MS/MS analyses. This diminished the interindividual variability among the stone formers that might affect kidney stone formation to different degrees. Second, this study examined only urinary proteins from stone formers without comparisons with those derived from nonstone subjects. Thus, this study characterized correlations within stone-former fractions rather than identifying differences between disease and nondisease states. Third, fractionation of urinary proteins by diethylaminoethyl anion exchange followed by FPLC using the GigaCap Q-650M column might lead to protein loss, especially those with strong positive charges and low-abundance proteins that might critically affect kidney stone formation processes. Fourth, the in vitro crystal assays, while allowing for individual kidney stone formation processes to be studied, might not fully represent the actual in vivo processes. Hence, the clinical implication of our large dataset needs careful interpretation. Fifth, the negative control used herein was a purified protein that was not processed identically as for all the chromatographic fractions. Therefore, the effects from sample preparation, if any, might affect the observations reported herein. Sixth, several physicochemical properties examined relied mainly on computational prediction, not on direct experimental results. Thus, the true properties of these proteins, e.g., calcium and/or oxalate ion binding, might be imprecise. Finally, the current status of stone formers as well as their stone size and recurrence were not analyzed. Therefore, the findings reported herein might reflect only the correlations between physicochemical properties of urinary proteins and their promoting activities on CaOx crystal aggregation and crystal–cell adhesion but not the actual causation of the disease.

## Conclusions

KSD is a multifaceted and intricate disease that can be affected by numerous factors, which can either facilitate or prevent the development of kidney stones. Herein, urinary proteins derived from patients with CaOx kidney stones were fractionated by FPLC using a GigaCap Q-650M column, and the protein fractions were investigated by CaOx crystal assays and identified by MS/MS analyses. From all 9 fractions (SFQ1 to SFQ9), almost all of them promoted CaOx crystallization, growth, aggregation, and crystal–cell adhesion, and 10, 12, 71, 71, 60, 55, 25, 38, and 6 were identified from SFQ1 to SFQ9, respectively. Among several abundance-weighted physicochemical parameters, MW, instability index, amino acid composition, and secondary structure positively correlated with abundance-weighted crystal aggregation-promoting activity and crystal adhesion-promoting activity. Ultimately, these findings elucidated the roles of urinary proteins from stone formers in kidney stone promotion, contributing to a better understanding of disease mechanisms.

## Data Availability

All data generated or analyzed during this study are included in this published article and Supplementary Materials and are also available from the corresponding author on reasonable request.
