# Supplementary material for: Promoting Effects of Urinary Proteins from Stone Formers and Influence of Their Physicochemical Properties on Calcium Oxalate Kidney Stone Formation
Source: Comput Struct Biotechnol J. 2026 May 14;35(1):0094. doi: 10.34133/csbj.0094 (PMC13172586; doi:10.34133/csbj.0094)
Supplement: Supplementary 1 — Supplementary Methods Table S1 [file csbj.0094.f1.zip › SFurine_GigaQ2_SupplTableS1_R1.pdf]

Promoting effects of urinary proteins from stone formers and influence of their physicochemical properties on calcium oxalate kidney stone formation

*Suttipong Suttapitugsakul, Paleerath Peerapen, and Visith Thongboonkerd**
*Correspondence to: *thongboonkerd@dr.com* (or) *vthongbo@yahoo.com*

Supplementary Table S1: Summary of urinary proteins identified in each protein fraction.

					Abundance-weighted crystal-promoting activity (%) ‡				Abundance-weighted physicochemical property δ													
									Calcium-/Oxalate-binding potential					Amino acid composition (%)					Secondary structure (%)			
**Fraction no.**	**UniProt ID**	**Protein name**	**Gene symbol**	**Relative protein abundance †**	**Crystallization**	**Crystal growth**	**Crystal aggregation**	**Crystal adhesion**	**No. of calcium-binding sites/protein**	**No. of oxalate-binding sites/protein**	**MW**	**Instability index**	**GRAVY**	**Aromatic amino acids**	**Polar amino acids**	**Non-polar amino acids**	**positive charged amino acids**	**Negative charged amino acids**	**Alpha helix**	**Extended strand**	**Beta turn**	**Random coil**
SFQ1	Q8N5M1	ATP synthase mitochondrial F1 complex assembly factor 2	*ATPAF2*	0.013	0.04	0.57	2.27	0.59	0.00	0.04	0.41	0.67	0.00	0.10	0.33	0.53	0.15	0.15	0.66	0.09	0.02	0.49
	P27708	CAD protein	*CAD*	0.013	0.04	0.57	2.27	0.59	0.00	0.20	3.05	0.48	0.00	0.09	0.30	0.61	0.12	0.14	0.46	0.20	0.08	0.52
	O75618	Death effector domain-containing protein	*DEDD*	0.105	0.30	4.75	18.98	4.95	0.00	0.32	3.87	4.72	-0.06	0.73	2.51	4.23	1.65	1.39	4.56	0.50	0.16	5.29
	Q8WWL7	G2/mitotic-specific cyclin-B3	*CCNB3*	0.013	0.04	0.57	2.27	0.59	0.00	0.01	1.98	0.74	-0.01	0.08	0.36	0.46	0.17	0.19	0.42	0.05	0.00	0.77
	O43390	Heterogeneous nuclear ribonucleoprotein R	*HNRNPR*	0.073	0.21	3.28	13.10	3.42	0.00	0.07	5.14	2.80	-0.07	0.72	1.41	3.02	1.07	1.03	2.18	0.71	0.27	4.09

	P02760	Protein AMBP	*AMBP*	0.013	0.04	0.57	2.27	0.59	0.00	0.06	0.49	0.62	0.00	0.12	0.33	0.53	0.13	0.14	0.24	0.19	0.03	0.79
	Q86SQ3	Putative adhesion G protein-coupled receptor E4P	*ADGRE4P*	0.013	0.04	0.57	2.27	0.59	0.01	0.04	0.64	0.45	0.01	0.15	0.37	0.56	0.11	0.08	0.49	0.26	0.05	0.45
	P0CB33	Putative zinc finger protein 735	*ZNF735*	0.337	0.98	15.24	60.84	15.86	0.00	0.67	16.02	17.52	-0.31	2.78	12.01	9.48	5.97	3.43	3.68	3.02	2.04	24.93
	Q9C0H5	Rho GTPase-activating protein 39	*ARHGAP39*	0.410	1.19	18.58	74.17	19.33	0.00	2.05	49.78	26.32	-0.27	3.45	10.73	17.17	4.85	4.85	9.13	1.21	0.34	30.36
	Q9NZV5	Selenoprotein N	*SELENON*	0.013	0.04	0.57	2.27	0.59	0.01	0.08	0.83	0.78	0.00	0.12	0.32	0.56	0.10	0.14	0.50	0.16	0.04	0.55
SFQ2	Q9P2J9	[Pyruvate dehydrogenase [acetyl-transferring]]-phosphatase 2, mitochondrial	*PDP2*	0.002	0.03	0.10	0.39	0.08	0.00	0.01	0.09	0.08	0.00	0.01	0.04	0.07	0.01	0.02	0.06	0.02	0.01	0.07
	Q9BXS0	Collagen alpha-1(XXV) chain	*COL25A1*	0.029	0.49	1.88	7.35	1.45	0.00	0.00	1.86	0.71	-0.03	0.04	0.41	1.75	0.34	0.32	0.23	0.03	0.04	2.56
	Q15375	Ephrin type-A receptor 7	*EPHA7*	0.002	0.03	0.10	0.39	0.08	0.00	0.01	0.17	0.06	0.00	0.01	0.04	0.06	0.02	0.02	0.04	0.03	0.01	0.08
	P55884	Eukaryotic translation initiation factor 3 subunit B	*EIF3B*	0.002	0.03	0.10	0.39	0.08	0.00	0.01	0.14	0.07	0.00	0.01	0.03	0.06	0.02	0.03	0.04	0.03	0.01	0.07
	A9QM74	Importin subunit alpha-8	*KPNA7*	0.064	1.10	4.20	16.43	3.25	0.00	0.26	3.65	3.54	0.00	0.30	1.71	3.05	0.62	0.72	4.00	0.16	0.24	2.01
	Q5T7N3	KN motif and ankyrin repeat domain-containing protein 4	*KANK4*	0.028	0.48	1.85	7.23	1.43	0.00	0.17	3.03	1.65	-0.02	0.11	0.82	1.25	0.24	0.39	0.95	0.06	0.11	1.69
	Q8TC05	Nuclear protein MDM1	*MDM1*	0.002	0.03	0.10	0.39	0.08	0.00	0.01	0.12	0.09	0.00	0.01	0.04	0.05	0.02	0.02	0.01	0.00	0.00	0.14
	Q9H4Q3	PR domain zinc finger protein 13	*PRDM13*	0.030	0.52	1.98	7.77	1.54	0.00	0.18	2.24	1.39	-0.01	0.20	0.63	1.60	0.33	0.27	0.27	0.15	0.02	2.60
	P07602	Prosaposin	*PSAP*	0.030	0.51	1.95	7.64	1.51	0.00	0.06	1.73	1.42	0.00	0.16	0.79	1.33	0.29	0.41	1.76	0.02	0.06	1.13

	Q8NA61	Protein chibby homolog 2	*CBY2*	0.019	0.32	1.23	4.82	0.95	0.00	0.08	0.97	1.51	-0.02	0.10	0.50	0.73	0.27	0.28	1.48	0.06	0.01	0.34
	Q9BPW5	Ras-like protein family member 11B	*RASL11B*	0.677	11.57	44.35	173.62	34.34	0.00	0.68	18.62	28.68	-0.09	4.09	20.20	30.03	7.92	5.46	23.75	10.37	2.46	31.12
	Q9C0H5	Rho GTPase-activating protein 39	*ARHGAP39*	0.117	2.00	7.68	30.06	5.95	0.00	0.59	14.22	7.52	-0.08	0.98	3.06	4.90	1.39	1.39	2.61	0.35	0.10	8.67
SFQ3	P63261	Actin, cytoplasmic 2	*ACTG1*	0.002	-0.03	-0.02	0.30	0.09	0.00	0.00	0.07	0.06	0.00	0.01	0.04	0.08	0.02	0.02	0.06	0.03	0.01	0.06
	P02763	Alpha-1-acid glycoprotein 1	*ORM1*	0.002	-0.04	-0.03	0.41	0.13	0.00	0.00	0.05	0.09	0.00	0.03	0.06	0.08	0.03	0.04	0.07	0.05	0.00	0.11
	P01011	Alpha-1-antichymotrypsin	*SERPINA3*	0.002	-0.04	-0.03	0.42	0.13	0.00	0.00	0.11	0.10	0.00	0.02	0.06	0.10	0.02	0.03	0.10	0.04	0.01	0.09
	P01009	Alpha-1-antitrypsin	*SERPINA1*	0.003	-0.05	-0.03	0.46	0.14	0.00	0.01	0.12	0.08	0.00	0.02	0.07	0.11	0.03	0.03	0.11	0.04	0.01	0.09
	P02765	Alpha-2-HS-glycoprotein	*AHSG*	0.005	-0.09	-0.06	0.91	0.29	0.00	0.01	0.20	0.24	0.00	0.03	0.14	0.25	0.04	0.06	0.09	0.07	0.01	0.35
	P05067	Amyloid-beta precursor protein	*APP*	0.000	0.00	0.00	0.02	0.01	0.00	0.00	0.01	0.01	0.00	0.00	0.00	0.00	0.00	0.00	0.00	0.00	0.00	0.01
	P05090	Apolipoprotein D	*APOD*	0.011	-0.19	-0.13	1.90	0.60	0.00	0.01	0.23	0.44	0.00	0.11	0.28	0.49	0.08	0.11	0.23	0.24	0.04	0.56
	P08519	Apolipoprotein(a)	*LPA*	0.001	-0.01	-0.01	0.14	0.05	0.00	0.00	0.18	0.04	0.00	0.01	0.03	0.03	0.01	0.01	0.00	0.01	0.00	0.06
	P98160	Basement membrane-specific heparan sulfate proteoglycan core protein	*HSPG2*	0.002	-0.04	-0.03	0.41	0.13	0.00	0.05	1.09	0.11	0.00	0.01	0.07	0.11	0.02	0.02	0.01	0.06	0.01	0.14
	P16278	Beta-galactosidase	*GLB1*	0.001	-0.01	-0.01	0.11	0.03	0.00	0.00	0.05	0.03	0.00	0.01	0.01	0.03	0.01	0.01	0.01	0.01	0.00	0.03
	P19835	Bile salt-activated lipase	*CEL*	0.000	-0.01	0.00	0.07	0.02	0.00	0.00	0.03	0.02	0.00	0.00	0.01	0.02	0.00	0.00	0.01	0.00	0.00	0.02

Q8WVV5	Butyrophilin subfamily 2 member A2	*BTN2A2*	0.000	-0.01	0.00	0.06	0.02	0.00	0.00	0.02	0.02	0.00	0.00	0.01	0.02	0.00	0.00	0.01	0.01	0.00	0.02
P0DP25	Calmodulin-3	*CALM3*	0.001	-0.01	-0.01	0.11	0.03	0.00	0.00	0.01	0.02	0.00	0.00	0.01	0.02	0.01	0.02	0.04	0.00	0.01	0.01
P16870	Carboxypeptidase E	*CPE*	0.000	0.00	0.00	0.03	0.01	0.00	0.00	0.01	0.01	0.00	0.00	0.00	0.01	0.00	0.00	0.00	0.00	0.00	0.01
P22792	Carboxypeptidase N subunit 2	*CPN2*	0.001	-0.01	-0.01	0.09	0.03	0.00	0.00	0.03	0.02	0.00	0.00	0.02	0.02	0.00	0.00	0.02	0.01	0.00	0.02
P16070	CD44 antigen	*CD44*	0.055	-0.99	-0.67	9.80	3.10	0.00	0.17	4.49	2.19	-0.04	0.39	2.14	1.86	0.40	0.71	0.44	0.06	0.01	5.00
Q9BY67	Cell adhesion molecule 1	*CADM1*	0.000	-0.01	0.00	0.05	0.02	0.00	0.00	0.01	0.01	0.00	0.00	0.01	0.01	0.00	0.00	0.00	0.01	0.00	0.02
P00450	Ceruloplasmin	*CP*	0.001	-0.03	-0.02	0.26	0.08	0.00	0.01	0.18	0.06	0.00	0.02	0.04	0.06	0.01	0.02	0.02	0.03	0.01	0.09
Q6UVK1	Chondroitin sulfate proteoglycan 4	*CSPG4*	0.003	-0.05	-0.04	0.53	0.17	0.00	0.06	0.74	0.13	0.00	0.02	0.07	0.15	0.02	0.03	0.02	0.10	0.01	0.17
P10909	Clusterin	*CLU*	0.003	-0.05	-0.03	0.47	0.15	0.00	0.00	0.14	0.14	0.00	0.02	0.08	0.09	0.03	0.04	0.17	0.02	0.00	0.07
P12109	Collagen alpha-1(VI) chain	*COL6A1*	0.001	-0.02	-0.01	0.20	0.06	0.00	0.00	0.12	0.03	0.00	0.01	0.02	0.05	0.01	0.01	0.03	0.01	0.00	0.07
P39059	Collagen alpha-1(XV) chain	*COL15A1*	0.004	-0.06	-0.04	0.63	0.20	0.00	0.01	0.50	0.14	0.00	0.02	0.07	0.20	0.03	0.04	0.02	0.02	0.00	0.31
P39060	Collagen alpha-1(XVIII) chain	*COL18A1*	0.002	-0.03	-0.02	0.32	0.10	0.00	0.02	0.32	0.09	0.00	0.01	0.04	0.11	0.01	0.02	0.01	0.01	0.00	0.15
P0C0L5	Complement C4-B	*C4B*	0.001	-0.01	-0.01	0.12	0.04	0.00	0.01	0.13	0.03	0.00	0.01	0.02	0.03	0.01	0.01	0.01	0.02	0.00	0.03
P24855	Deoxyribonuclease-1	*DNASE1*	0.001	-0.01	-0.01	0.11	0.04	0.00	0.00	0.02	0.02	0.00	0.01	0.01	0.03	0.00	0.01	0.02	0.02	0.00	0.03

Q9HCU0	Endosialin	*CD248*	0.013	-0.23	-0.16	2.28	0.72	0.01	0.04	1.04	0.75	0.00	0.08	0.36	0.63	0.08	0.13	0.07	0.15	0.02	1.04
P08294	Extracellular superoxide dismutase [Cu-Zn]	*SOD3*	0.001	-0.02	-0.01	0.15	0.05	0.00	0.00	0.02	0.04	0.00	0.01	0.02	0.04	0.01	0.01	0.01	0.02	0.00	0.06
P35555	Fibrillin-1	*FBN1*	0.006	-0.11	-0.08	1.13	0.36	0.27	0.04	1.98	0.28	0.00	0.04	0.23	0.23	0.05	0.08	0.00	0.05	0.02	0.56
P02671	Fibrinogen alpha chain	*FGA*	0.029	-0.52	-0.35	5.11	1.62	0.03	0.17	2.73	1.17	-0.02	0.24	0.91	1.03	0.32	0.38	0.66	0.20	0.10	1.91
P02751	Fibronectin	*FN1*	0.002	-0.04	-0.03	0.38	0.12	0.00	0.01	0.58	0.09	0.00	0.02	0.07	0.09	0.02	0.02	0.00	0.06	0.01	0.14
P98095	Fibulin-2	*FBLN2*	0.001	-0.03	-0.02	0.25	0.08	0.01	0.01	0.18	0.09	0.00	0.01	0.04	0.06	0.01	0.02	0.01	0.02	0.01	0.11
Q08380	Galectin-3-binding protein	*LGALS3BP*	0.008	-0.15	-0.10	1.49	0.47	0.00	0.02	0.55	0.32	0.00	0.10	0.23	0.35	0.07	0.09	0.17	0.15	0.03	0.49
Q8NBJ4	Golgi membrane protein 1	*GOLM1*	0.003	-0.06	-0.04	0.59	0.19	0.00	0.02	0.15	0.18	0.00	0.01	0.09	0.13	0.04	0.06	0.21	0.00	0.00	0.12
P01876	Immunoglobulin heavy constant alpha 1	*IGHA1*	0.002	-0.03	-0.02	0.34	0.11	0.00	0.00	0.08	0.11	0.00	0.01	0.06	0.08	0.01	0.02	0.02	0.05	0.00	0.12
P01834	Immunoglobulin kappa constant	*IGKC*	0.002	-0.04	-0.03	0.37	0.12	0.00	0.00	0.02	0.12	0.00	0.02	0.08	0.07	0.02	0.02	0.02	0.06	0.01	0.12
P0DOY3	Immunoglobulin lambda constant 3	*IGLC3*	0.001	-0.03	-0.02	0.26	0.08	0.00	0.00	0.02	0.11	0.00	0.01	0.05	0.05	0.01	0.01	0.01	0.04	0.01	0.08
O95998	Interleukin-18-binding protein	*IL18BP*	0.001	-0.02	-0.02	0.22	0.07	0.00	0.00	0.03	0.06	0.00	0.01	0.04	0.06	0.01	0.01	0.02	0.02	0.00	0.08
P06870	Kallikrein-1	*KLK1*	0.045	-0.81	-0.55	8.06	2.55	0.00	0.05	1.31	1.86	-0.01	0.40	1.31	1.99	0.24	0.59	0.73	1.11	0.41	2.28
P01042	Kininogen-1	*KNG1*	0.029	-0.52	-0.36	5.20	1.65	0.00	0.06	2.10	1.20	-0.02	0.22	0.99	1.02	0.33	0.37	0.43	0.34	0.02	2.13

P98164	Low-density lipoprotein receptor-related protein 2	*LRP2*	0.008	-0.15	-0.10	1.48	0.47	0.01	0.19	4.35	0.31	0.00	0.08	0.27	0.30	0.07	0.12	0.03	0.21	0.04	0.56
P10253	Lysosomal alpha-glucosidase	*GAA*	0.000	-0.01	-0.01	0.09	0.03	0.00	0.00	0.05	0.02	0.00	0.00	0.01	0.02	0.00	0.00	0.01	0.01	0.00	0.03
P09603	Macrophage colony-stimulating factor 1	*CSF1*	0.003	-0.06	-0.04	0.55	0.17	0.00	0.00	0.19	0.22	0.00	0.02	0.09	0.13	0.03	0.04	0.06	0.01	0.00	0.23
O00187	Mannan-binding lectin serine protease 2	*MASP2*	0.001	-0.02	-0.01	0.16	0.05	0.00	0.00	0.07	0.04	0.00	0.01	0.02	0.04	0.01	0.01	0.01	0.02	0.00	0.06
P08571	Monocyte differentiation antigen CD14	*CD14*	0.003	-0.06	-0.04	0.55	0.18	0.00	0.02	0.12	0.13	0.00	0.01	0.08	0.16	0.03	0.03	0.14	0.02	0.00	0.15
Q13477	Mucosal addressin cell adhesion molecule 1	*MADCAM1*	0.000	-0.01	0.00	0.06	0.02	0.00	0.00	0.01	0.02	0.00	0.00	0.01	0.02	0.00	0.00	0.00	0.01	0.00	0.02
Q99574	Neuroserpin	*SERPINI1*	0.000	0.00	0.00	0.02	0.01	0.00	0.00	0.01	0.00	0.00	0.00	0.00	0.01	0.00	0.00	0.00	0.00	0.00	0.01
P14543	Nidogen-1	*NID1*	0.006	-0.11	-0.07	1.06	0.34	0.01	0.04	0.81	0.27	0.00	0.05	0.18	0.24	0.05	0.07	0.02	0.10	0.02	0.46
P10153	Non-secretory ribonuclease	*RNASE2*	0.000	0.00	0.00	0.04	0.01	0.00	0.00	0.00	0.01	0.00	0.00	0.01	0.01	0.00	0.00	0.01	0.00	0.00	0.01
P10451	Osteopontin	*SPP1*	0.019	-0.33	-0.23	3.30	1.04	0.00	0.00	0.66	0.99	-0.02	0.11	0.60	0.53	0.17	0.44	0.48	0.13	0.00	1.24
O95497	Pantetheinase	*VNN1*	0.000	-0.01	-0.01	0.08	0.03	0.00	0.00	0.03	0.02	0.00	0.01	0.01	0.02	0.00	0.00	0.01	0.01	0.00	0.03
P0DJD9	Pepsin A-5	*PGA5*	0.003	-0.06	-0.04	0.60	0.19	0.00	0.00	0.14	0.15	0.00	0.04	0.10	0.15	0.01	0.03	0.06	0.10	0.02	0.16
Q6UXB8	Peptidase inhibitor 16	*PI16*	0.007	-0.13	-0.09	1.33	0.42	0.00	0.02	0.37	0.40	0.00	0.04	0.21	0.36	0.06	0.09	0.10	0.04	0.00	0.61
Q96FE7	Phosphoinositide-3-kinase-interacting protein 1	*PIK3IP1*	0.026	-0.46	-0.32	4.60	1.46	0.00	0.00	0.73	0.87	-0.01	0.16	0.65	1.27	0.21	0.30	0.56	0.25	0.10	1.68

P05155	Plasma protease C1 inhibitor	*SERPING1*	0.003	-0.05	-0.04	0.53	0.17	0.00	0.01	0.16	0.10	0.00	0.02	0.10	0.12	0.03	0.03	0.11	0.04	0.01	0.14
Q9HCN6	Platelet glycoprotein VI	*GP6*	0.001	-0.01	-0.01	0.12	0.04	0.00	0.00	0.03	0.04	0.00	0.01	0.02	0.03	0.01	0.01	0.01	0.01	0.00	0.05
P01133	Pro-epidermal growth factor	*EGF*	0.016	-0.28	-0.19	2.82	0.89	0.05	0.06	2.13	0.77	0.00	0.12	0.46	0.66	0.15	0.20	0.05	0.34	0.05	1.16
P28799	Progranulin	*GRN*	0.000	-0.01	-0.01	0.09	0.03	0.00	0.00	0.03	0.02	0.00	0.00	0.02	0.02	0.00	0.00	0.00	0.00	0.00	0.05
P07602	Prosaposin	*PSAP*	0.041	-0.74	-0.51	7.36	2.33	0.00	0.08	2.40	1.97	0.00	0.22	1.10	1.85	0.40	0.57	2.45	0.03	0.09	1.57
P02760	Protein AMBP	*AMBP*	0.517	-9.29	-6.34	92.08	29.15	0.00	2.59	20.18	25.40	-0.15	5.00	13.52	21.90	5.44	5.88	9.70	7.94	1.32	32.77
Q8N114	Protein shisa-5	*SHISA5*	0.001	-0.01	-0.01	0.10	0.03	0.00	0.00	0.01	0.04	0.00	0.01	0.01	0.03	0.00	0.00	0.01	0.01	0.00	0.04
P00734	Prothrombin	*F2*	0.004	-0.08	-0.05	0.76	0.24	0.00	0.02	0.30	0.18	0.00	0.04	0.11	0.17	0.05	0.06	0.07	0.04	0.02	0.30
P22105	Tenascin-X	*TNXB*	0.000	0.00	0.00	0.04	0.01	0.00	0.01	0.11	0.01	0.00	0.00	0.01	0.01	0.00	0.00	0.00	0.01	0.00	0.02
P07204	Thrombomodulin	*THBD*	0.001	-0.02	-0.01	0.15	0.05	0.00	0.00	0.05	0.04	0.00	0.01	0.02	0.04	0.00	0.01	0.01	0.01	0.00	0.06
P08138	Tumor necrosis factor receptor superfamily member 16	*NGFR*	0.003	-0.05	-0.04	0.53	0.17	0.00	0.01	0.13	0.17	0.00	0.01	0.09	0.13	0.02	0.04	0.05	0.02	0.01	0.21
Q9GZX9	Twisted gastrulation protein homolog 1	*TWSG1*	0.001	-0.02	-0.01	0.18	0.06	0.00	0.00	0.03	0.06	0.00	0.01	0.04	0.04	0.01	0.01	0.03	0.01	0.00	0.06
P30530	Tyrosine-protein kinase receptor UFO	*AXL*	0.011	-0.20	-0.13	1.95	0.62	0.00	0.03	1.08	0.58	0.00	0.08	0.28	0.52	0.09	0.13	0.19	0.19	0.03	0.70
P07911	Uromodulin	*UMOD*	0.063	-1.13	-0.77	11.22	3.55	0.13	0.19	4.40	2.55	-0.01	0.54	2.11	2.52	0.45	0.68	0.03	1.13	0.04	5.10

	Q6EMK4	Vasorin	*VASN*	0.003	-0.06	-0.04	0.60	0.19	0.00	0.03	0.24	0.19	0.00	0.01	0.08	0.18	0.03	0.03	0.10	0.04	0.01	0.19
	P02774	Vitamin D-binding protein	*GC*	0.004	-0.07	-0.05	0.70	0.22	0.00	0.00	0.21	0.17	0.00	0.03	0.12	0.14	0.05	0.06	0.28	0.01	0.01	0.11
	P22891	Vitamin K-dependent protein Z	*PROZ*	0.003	-0.06	-0.04	0.56	0.18	0.00	0.01	0.14	0.13	0.00	0.02	0.09	0.13	0.03	0.04	0.07	0.06	0.02	0.15
	P04004	Vitronectin	*VTN*	0.003	-0.05	-0.03	0.49	0.16	0.00	0.01	0.15	0.13	0.00	0.03	0.07	0.11	0.03	0.04	0.02	0.04	0.02	0.21
SFQ4	P63261	Actin, cytoplasmic 2	*ACTG1*	0.004	-0.06	0.10	1.30	0.20	0.00	0.01	0.17	0.15	0.00	0.03	0.09	0.18	0.04	0.05	0.15	0.09	0.03	0.15
	P16112	Aggrecan core protein	*ACAN*	0.000	-0.01	0.01	0.13	0.02	0.00	0.00	0.10	0.02	0.00	0.00	0.01	0.02	0.00	0.01	0.00	0.00	0.00	0.04
	P01011	Alpha-1-antichymotrypsin	*SERPINA3*	0.002	-0.04	0.06	0.76	0.12	0.00	0.00	0.11	0.10	0.00	0.02	0.06	0.10	0.02	0.03	0.10	0.04	0.01	0.09
	P01009	Alpha-1-antitrypsin	*SERPINA1*	0.002	-0.03	0.04	0.60	0.09	0.00	0.00	0.09	0.06	0.00	0.02	0.05	0.08	0.02	0.03	0.08	0.03	0.01	0.07
	P02765	Alpha-2-HS-glycoprotein	*AHSG*	0.004	-0.06	0.09	1.29	0.20	0.00	0.00	0.16	0.19	0.00	0.02	0.11	0.20	0.03	0.05	0.07	0.06	0.01	0.27
	P05067	Amyloid-beta precursor protein	*APP*	0.000	0.00	0.01	0.08	0.01	0.00	0.00	0.02	0.01	0.00	0.00	0.01	0.01	0.00	0.00	0.01	0.00	0.00	0.01
	P05090	Apolipoprotein D	*APOD*	0.004	-0.06	0.09	1.27	0.20	0.00	0.00	0.08	0.16	0.00	0.04	0.10	0.18	0.03	0.04	0.09	0.09	0.01	0.21
	P08519	Apolipoprotein(a)	*LPA*	0.001	-0.01	0.01	0.17	0.03	0.00	0.00	0.12	0.02	0.00	0.01	0.02	0.02	0.00	0.00	0.00	0.01	0.00	0.04
	P98160	Basement membrane-specific heparan sulfate proteoglycan core protein	*HSPG2*	0.004	-0.06	0.10	1.36	0.21	0.00	0.09	1.99	0.20	0.00	0.02	0.14	0.19	0.03	0.04	0.02	0.11	0.02	0.26
	P16278	Beta-galactosidase	*GLB1*	0.001	-0.02	0.03	0.36	0.06	0.00	0.00	0.09	0.05	0.00	0.01	0.03	0.05	0.01	0.01	0.02	0.03	0.01	0.06

P13727	Bone marrow proteoglycan	*PRG2*	0.001	-0.01	0.02	0.25	0.04	0.00	0.00	0.02	0.04	0.00	0.01	0.02	0.03	0.01	0.01	0.01	0.01	0.00	0.05
Q8WVV5	Butyrophilin subfamily 2 member A2	*BTN2A2*	0.000	0.00	0.00	0.07	0.01	0.00	0.00	0.01	0.01	0.00	0.00	0.00	0.01	0.00	0.00	0.00	0.01	0.00	0.01
P0DP25	Calmodulin-3	*CALM3*	0.003	-0.04	0.06	0.80	0.13	0.01	0.00	0.04	0.07	0.00	0.02	0.05	0.10	0.02	0.06	0.16	0.01	0.02	0.06
P22792	Carboxypeptidase N subunit 2	*CPN2*	0.000	0.00	0.01	0.09	0.01	0.00	0.00	0.02	0.01	0.00	0.00	0.01	0.01	0.00	0.00	0.01	0.00	0.00	0.01
P16070	CD44 antigen	*CD44*	0.054	-0.82	1.27	17.36	2.71	0.00	0.16	4.42	2.15	-0.04	0.38	2.11	1.84	0.39	0.70	0.43	0.06	0.01	4.92
Q9BY67	Cell adhesion molecule 1	*CADM1*	0.001	-0.01	0.01	0.18	0.03	0.00	0.00	0.03	0.02	0.00	0.00	0.02	0.03	0.00	0.01	0.01	0.02	0.00	0.03
Q6UVK1	Chondroitin sulfate proteoglycan 4	*CSPG4*	0.006	-0.09	0.14	1.95	0.30	0.00	0.12	1.52	0.27	0.00	0.04	0.15	0.30	0.05	0.07	0.04	0.20	0.02	0.34
P10909	Clusterin	*CLU*	0.002	-0.03	0.05	0.71	0.11	0.00	0.00	0.12	0.12	0.00	0.02	0.07	0.08	0.03	0.03	0.15	0.02	0.00	0.06
Q6UXG3	CMRF35-like molecule 9	*CD300LG*	0.001	-0.01	0.02	0.24	0.04	0.00	0.00	0.03	0.04	0.00	0.00	0.02	0.03	0.01	0.01	0.01	0.01	0.00	0.05
P39059	Collagen alpha-1(XV) chain	*COL15A1*	0.005	-0.08	0.12	1.71	0.27	0.00	0.02	0.76	0.21	0.00	0.03	0.11	0.30	0.04	0.06	0.03	0.03	0.01	0.46
P0C0L5	Complement C4-B	*C4B*	0.001	-0.01	0.02	0.21	0.03	0.00	0.01	0.13	0.03	0.00	0.01	0.02	0.03	0.01	0.01	0.01	0.02	0.00	0.03
P24855	Deoxyribonuclease-1	*DNASE1*	0.001	-0.01	0.02	0.27	0.04	0.00	0.00	0.03	0.03	0.00	0.01	0.02	0.04	0.01	0.01	0.03	0.02	0.00	0.04
P36957	Dihydrolipoyllysine-residue succinyltransferase component of 2-oxoglutarate dehydrogenase complex, mitochondrial	*DLST*	0.000	-0.01	0.01	0.12	0.02	0.00	0.00	0.02	0.02	0.00	0.00	0.01	0.02	0.00	0.00	0.01	0.01	0.00	0.02
P53634	Dipeptidyl peptidase 1	*CTSC*	0.000	0.00	0.01	0.07	0.01	0.00	0.00	0.01	0.01	0.00	0.00	0.01	0.01	0.00	0.00	0.01	0.00	0.00	0.01

Q9HCU0	Endosialin	*CD248*	0.008	-0.11	0.18	2.43	0.38	0.01	0.02	0.61	0.45	0.00	0.05	0.21	0.37	0.05	0.08	0.04	0.09	0.01	0.62
P56537	Eukaryotic translation initiation factor 6	*EIF6*	0.000	-0.01	0.01	0.13	0.02	0.00	0.00	0.01	0.01	0.00	0.00	0.01	0.02	0.00	0.01	0.01	0.01	0.00	0.01
P08294	Extracellular superoxide dismutase [Cu-Zn]	*SOD3*	0.001	-0.02	0.03	0.40	0.06	0.00	0.00	0.03	0.06	0.00	0.01	0.03	0.06	0.01	0.02	0.01	0.03	0.00	0.08
P35555	Fibrillin-1	*FBN1*	0.004	-0.05	0.08	1.16	0.18	0.16	0.02	1.13	0.16	0.00	0.02	0.13	0.13	0.03	0.05	0.00	0.03	0.01	0.32
P02671	Fibrinogen alpha chain	*FGA*	0.023	-0.34	0.53	7.27	1.13	0.02	0.14	2.16	0.93	-0.02	0.19	0.72	0.81	0.25	0.30	0.52	0.16	0.08	1.51
P02751	Fibronectin	*FN1*	0.001	-0.01	0.02	0.23	0.04	0.00	0.00	0.19	0.03	0.00	0.01	0.02	0.03	0.01	0.01	0.00	0.02	0.00	0.05
P98095	Fibulin-2	*FBLN2*	0.001	-0.01	0.01	0.18	0.03	0.01	0.00	0.07	0.03	0.00	0.00	0.02	0.02	0.00	0.01	0.01	0.01	0.00	0.04
Q08380	Galectin-3-binding protein	*LGALS3BP*	0.010	-0.15	0.24	3.25	0.51	0.00	0.02	0.66	0.40	0.00	0.12	0.28	0.42	0.08	0.11	0.20	0.18	0.03	0.60
Q8NBJ4	Golgi membrane protein 1	*GOLM1*	0.002	-0.03	0.05	0.64	0.10	0.00	0.01	0.09	0.11	0.00	0.01	0.05	0.08	0.03	0.04	0.13	0.00	0.00	0.07
Q04756	Hepatocyte growth factor activator	*HGFAC*	0.000	0.00	0.01	0.09	0.01	0.00	0.00	0.02	0.01	0.00	0.00	0.01	0.01	0.00	0.00	0.00	0.00	0.00	0.02
P01876	Immunoglobulin heavy constant alpha 1	*IGHA1*	0.000	0.00	0.00	0.04	0.01	0.00	0.00	0.00	0.01	0.00	0.00	0.00	0.00	0.00	0.00	0.00	0.00	0.00	0.01
P01834	Immunoglobulin kappa constant	*IGKC*	0.001	-0.01	0.02	0.28	0.04	0.00	0.00	0.01	0.05	0.00	0.01	0.03	0.03	0.01	0.01	0.01	0.02	0.01	0.05
P0DOY3	Immunoglobulin lambda constant 3	*IGLC3*	0.000	-0.01	0.01	0.13	0.02	0.00	0.00	0.00	0.03	0.00	0.00	0.01	0.01	0.00	0.00	0.00	0.01	0.00	0.02
O95998	Interleukin-18-binding protein	*IL18BP*	0.000	-0.01	0.01	0.14	0.02	0.00	0.00	0.01	0.02	0.00	0.00	0.02	0.02	0.00	0.00	0.01	0.01	0.00	0.03

P06870	Kallikrein-1	*KLK1*	0.087	-1.30	2.02	27.70	4.32	0.00	0.09	2.50	3.55	-0.01	0.76	2.51	3.80	0.46	1.12	1.39	2.12	0.79	4.36
P01042	Kininogen-1	*KNG1*	0.014	-0.22	0.33	4.58	0.71	0.00	0.03	1.03	0.59	-0.01	0.11	0.48	0.50	0.16	0.18	0.21	0.17	0.01	1.04
Q16363	Laminin subunit alpha-4	*LAMA4*	0.000	0.00	0.00	0.04	0.01	0.00	0.00	0.02	0.00	0.00	0.00	0.00	0.00	0.00	0.00	0.00	0.00	0.00	0.01
P98164	Low-density lipoprotein receptor-related protein 2	*LRP2*	0.004	-0.06	0.09	1.26	0.20	0.00	0.09	2.06	0.15	0.00	0.04	0.13	0.14	0.03	0.06	0.02	0.10	0.02	0.26
P09603	Macrophage colony-stimulating factor 1	*CSF1*	0.005	-0.07	0.11	1.46	0.23	0.00	0.00	0.28	0.33	0.00	0.02	0.14	0.20	0.04	0.06	0.09	0.02	0.01	0.33
P08571	Monocyte differentiation antigen CD14	*CD14*	0.001	-0.02	0.03	0.48	0.07	0.00	0.01	0.06	0.06	0.00	0.01	0.04	0.08	0.01	0.01	0.07	0.01	0.00	0.07
Q13477	Mucosal addressin cell adhesion molecule 1	*MADCAM1*	0.001	-0.01	0.02	0.29	0.04	0.00	0.00	0.04	0.06	0.00	0.00	0.03	0.05	0.01	0.01	0.01	0.02	0.00	0.07
P14543	Nidogen-1	*NID1*	0.009	-0.14	0.21	2.89	0.45	0.02	0.06	1.23	0.41	0.00	0.08	0.27	0.37	0.07	0.11	0.03	0.15	0.03	0.70
P10451	Osteopontin	*SPP1*	0.061	-0.91	1.42	19.44	3.03	0.00	0.00	2.15	3.24	-0.07	0.35	1.97	1.74	0.56	1.45	1.59	0.43	0.00	4.06
P0DJD9	Pepsin A-5	*PGA5*	0.003	-0.04	0.06	0.88	0.14	0.00	0.00	0.11	0.12	0.00	0.03	0.08	0.12	0.01	0.03	0.05	0.08	0.01	0.13
Q6UXB8	Peptidase inhibitor 16	*PI16*	0.008	-0.12	0.18	2.49	0.39	0.00	0.02	0.39	0.41	0.00	0.04	0.22	0.37	0.06	0.09	0.10	0.04	0.00	0.63
Q96FE7	Phosphoinositide-3-kinase-interacting protein 1	*PIK3IP1*	0.012	-0.18	0.28	3.81	0.59	0.00	0.00	0.34	0.40	0.00	0.07	0.30	0.58	0.10	0.14	0.26	0.11	0.05	0.77
P05155	Plasma protease C1 inhibitor	*SERPING1*	0.002	-0.03	0.04	0.57	0.09	0.00	0.00	0.10	0.06	0.00	0.01	0.06	0.07	0.02	0.02	0.06	0.02	0.01	0.08
Q9HCN6	Platelet glycoprotein VI	*GP6*	0.001	-0.01	0.02	0.23	0.04	0.00	0.00	0.03	0.04	0.00	0.01	0.02	0.03	0.01	0.01	0.01	0.01	0.00	0.05

P01133	Pro-epidermal growth factor	*EGF*	0.015	-0.23	0.35	4.86	0.76	0.05	0.06	2.03	0.74	0.00	0.12	0.44	0.63	0.14	0.19	0.04	0.32	0.04	1.11
P28799	Progranulin	*GRN*	0.001	-0.02	0.03	0.44	0.07	0.00	0.00	0.09	0.07	0.00	0.01	0.05	0.05	0.01	0.01	0.00	0.00	0.00	0.14
P07602	Prosaposin	*PSAP*	0.027	-0.41	0.64	8.71	1.36	0.00	0.05	1.58	1.29	0.00	0.15	0.72	1.22	0.26	0.37	1.61	0.02	0.06	1.03
P02760	Protein AMBP	*AMBP*	0.522	-7.86	12.20	167.03	26.03	0.00	2.61	20.36	25.63	-0.16	5.04	13.64	22.10	5.49	5.93	9.79	8.01	1.34	33.07
Q9UNF0	Protein kinase C and casein kinase substrate in neurons protein 2	*PACSIN2*	0.001	-0.01	0.01	0.16	0.03	0.00	0.00	0.03	0.02	0.00	0.00	0.01	0.02	0.01	0.01	0.03	0.00	0.00	0.02
Q7Z3E1	Protein mono-ADP-ribosyltransferase TIPARP	*TIPARP*	0.000	0.00	0.00	0.04	0.01	0.00	0.00	0.01	0.01	0.00	0.00	0.00	0.00	0.00	0.00	0.00	0.00	0.00	0.01
P00734	Prothrombin	*F2*	0.006	-0.09	0.14	1.98	0.31	0.01	0.02	0.43	0.25	0.00	0.06	0.16	0.24	0.07	0.09	0.10	0.06	0.02	0.44
Q14515	SPARC-like protein 1	*SPARCL1*	0.001	-0.02	0.02	0.32	0.05	0.00	0.01	0.08	0.05	0.00	0.01	0.03	0.03	0.01	0.02	0.04	0.00	0.00	0.06
P18827	Syndecan-1	*SDC1*	0.000	0.00	0.01	0.09	0.01	0.00	0.00	0.01	0.01	0.00	0.00	0.01	0.01	0.00	0.00	0.00	0.00	0.00	0.02
P31431	Syndecan-4	*SDC4*	0.000	0.00	0.01	0.08	0.01	0.00	0.00	0.01	0.01	0.00	0.00	0.00	0.01	0.00	0.00	0.01	0.00	0.00	0.02
P02766	Transthyretin	*TTR*	0.000	0.00	0.01	0.11	0.02	0.00	0.00	0.01	0.01	0.00	0.00	0.01	0.02	0.00	0.00	0.00	0.01	0.00	0.02
P04155	Trefoil factor 1	*TFF1*	0.000	0.00	0.00	0.04	0.01	0.00	0.00	0.00	0.01	0.00	0.00	0.00	0.01	0.00	0.00	0.00	0.00	0.00	0.01
P08138	Tumor necrosis factor receptor superfamily member 16	*NGFR*	0.001	-0.01	0.01	0.19	0.03	0.00	0.00	0.03	0.04	0.00	0.00	0.02	0.03	0.00	0.01	0.01	0.00	0.00	0.04
P30530	Tyrosine-protein kinase receptor UFO	*AXL*	0.006	-0.10	0.15	2.05	0.32	0.00	0.02	0.63	0.34	0.00	0.05	0.16	0.30	0.05	0.07	0.11	0.11	0.02	0.41

	P07911	Uromodulin	*UMOD*	0.053	-0.80	1.24	16.96	2.64	0.11	0.16	3.70	2.15	-0.01	0.46	1.77	2.12	0.38	0.57	0.02	0.95	0.03	4.29
	Q6EMK4	Vasorin	*VASN*	0.001	-0.01	0.02	0.30	0.05	0.00	0.01	0.07	0.05	0.00	0.00	0.02	0.05	0.01	0.01	0.03	0.01	0.00	0.05
	P22891	Vitamin K-dependent protein Z	*PROZ*	0.003	-0.04	0.07	0.90	0.14	0.00	0.01	0.13	0.12	0.00	0.02	0.08	0.11	0.03	0.03	0.07	0.06	0.02	0.14
	Q7Z5L0	Vitelline membrane outer layer protein 1 homolog	*VMO1*	0.000	-0.01	0.01	0.14	0.02	0.00	0.00	0.01	0.01	0.00	0.00	0.01	0.02	0.00	0.01	0.01	0.01	0.00	0.03
	P04004	Vitronectin	*VTN*	0.005	-0.08	0.13	1.75	0.27	0.00	0.01	0.30	0.26	0.00	0.06	0.13	0.21	0.06	0.08	0.04	0.07	0.03	0.41
SFQ5	P63261	Actin, cytoplasmic 2	*ACTG1*	0.003	-0.04	0.07	0.90	0.16	0.00	0.01	0.13	0.12	0.00	0.03	0.07	0.14	0.03	0.04	0.11	0.06	0.02	0.11
	P01011	Alpha-1-antichymotrypsin	*SERPINA3*	0.003	-0.04	0.06	0.85	0.15	0.00	0.01	0.14	0.12	0.00	0.03	0.07	0.13	0.03	0.04	0.12	0.05	0.01	0.11
	P01009	Alpha-1-antitrypsin	*SERPINA1*	0.002	-0.02	0.04	0.56	0.10	0.00	0.00	0.09	0.06	0.00	0.02	0.05	0.08	0.02	0.03	0.08	0.03	0.01	0.07
	P05090	Apolipoprotein D	*APOD*	0.001	-0.01	0.01	0.15	0.03	0.00	0.00	0.01	0.02	0.00	0.01	0.01	0.02	0.00	0.01	0.01	0.01	0.00	0.03
	P08519	Apolipoprotein(a)	*LPA*	0.002	-0.02	0.03	0.47	0.08	0.00	0.00	0.36	0.07	0.00	0.02	0.06	0.06	0.01	0.01	0.01	0.02	0.01	0.12
	P98160	Basement membrane-specific heparan sulfate proteoglycan core protein	*HSPG2*	0.001	-0.02	0.03	0.37	0.06	0.00	0.03	0.59	0.06	0.00	0.01	0.04	0.06	0.01	0.01	0.01	0.03	0.01	0.08
	P16278	Beta-galactosidase	*GLB1*	0.001	-0.02	0.03	0.41	0.07	0.00	0.01	0.11	0.06	0.00	0.02	0.03	0.06	0.01	0.01	0.03	0.03	0.01	0.07
	P06865	Beta-hexosaminidase subunit alpha	*HEXA*	0.000	0.00	0.00	0.06	0.01	0.00	0.00	0.01	0.01	0.00	0.00	0.00	0.01	0.00	0.00	0.01	0.00	0.00	0.01
	P19835	Bile salt-activated lipase	*CEL*	0.000	0.00	0.00	0.06	0.01	0.00	0.00	0.02	0.01	0.00	0.00	0.00	0.01	0.00	0.00	0.00	0.00	0.00	0.01

P43251	Biotinidase	*BTD*	0.000	0.00	0.01	0.07	0.01	0.00	0.00	0.01	0.01	0.00	0.00	0.01	0.01	0.00	0.00	0.01	0.01	0.00	0.01
P13727	Bone marrow proteoglycan	*PRG2*	0.000	-0.01	0.01	0.14	0.02	0.00	0.00	0.01	0.03	0.00	0.00	0.01	0.02	0.01	0.01	0.01	0.01	0.00	0.03
P16070	CD44 antigen	*CD44*	0.032	-0.40	0.70	9.51	1.64	0.00	0.10	2.65	1.29	-0.02	0.23	1.27	1.10	0.24	0.42	0.26	0.04	0.01	2.95
Q9BY67	Cell adhesion molecule 1	*CADM1*	0.001	-0.01	0.02	0.26	0.05	0.00	0.00	0.04	0.03	0.00	0.01	0.03	0.04	0.01	0.01	0.01	0.03	0.00	0.05
Q6UVK1	Chondroitin sulfate proteoglycan 4	*CSPG4*	0.001	-0.01	0.01	0.17	0.03	0.00	0.01	0.14	0.03	0.00	0.00	0.01	0.03	0.00	0.01	0.00	0.02	0.00	0.03
P10909	Clusterin	*CLU*	0.004	-0.05	0.08	1.14	0.20	0.00	0.00	0.21	0.21	0.00	0.03	0.12	0.13	0.05	0.06	0.26	0.03	0.00	0.10
P39059	Collagen alpha-1(XV) chain	*COL15A1*	0.001	-0.01	0.02	0.21	0.04	0.00	0.00	0.10	0.03	0.00	0.00	0.01	0.04	0.01	0.01	0.00	0.00	0.00	0.06
P01024	Complement C3	*C3*	0.000	0.00	0.00	0.06	0.01	0.00	0.00	0.04	0.01	0.00	0.00	0.01	0.01	0.00	0.00	0.00	0.01	0.00	0.01
P24855	Deoxyribonuclease-1	*DNASE1*	0.000	0.00	0.01	0.08	0.01	0.00	0.00	0.01	0.01	0.00	0.00	0.01	0.01	0.00	0.00	0.01	0.01	0.00	0.01
P27487	Dipeptidyl peptidase 4	*DPP4*	0.000	0.00	0.00	0.07	0.01	0.00	0.00	0.02	0.01	0.00	0.00	0.01	0.01	0.00	0.00	0.00	0.01	0.00	0.01
Q9HCU0	Endosialin	*CD248*	0.005	-0.06	0.10	1.35	0.23	0.00	0.01	0.37	0.27	0.00	0.03	0.13	0.23	0.03	0.05	0.03	0.05	0.01	0.37
P08294	Extracellular superoxide dismutase [Cu-Zn]	*SOD3*	0.001	-0.02	0.03	0.36	0.06	0.00	0.00	0.03	0.05	0.00	0.01	0.03	0.06	0.01	0.01	0.01	0.03	0.00	0.08
P35555	Fibrillin-1	*FBN1*	0.003	-0.03	0.06	0.75	0.13	0.11	0.02	0.80	0.11	0.00	0.02	0.09	0.09	0.02	0.03	0.00	0.02	0.01	0.23
P02671	Fibrinogen alpha chain	*FGA*	0.012	-0.15	0.26	3.53	0.61	0.01	0.07	1.15	0.49	-0.01	0.10	0.38	0.43	0.14	0.16	0.28	0.08	0.04	0.80

P02751	Fibronectin	*FN1*	0.002	-0.03	0.05	0.68	0.12	0.00	0.01	0.63	0.09	0.00	0.02	0.08	0.09	0.02	0.03	0.00	0.07	0.01	0.16
Q08380	Galectin-3-binding protein	*LGALS3BP*	0.026	-0.32	0.55	7.51	1.30	0.00	0.05	1.68	1.00	0.00	0.30	0.71	1.07	0.21	0.28	0.51	0.46	0.08	1.50
Q8NBJ4	Golgi membrane protein 1	*GOLM1*	0.003	-0.04	0.07	0.91	0.16	0.00	0.02	0.14	0.17	0.00	0.01	0.08	0.12	0.04	0.05	0.20	0.00	0.00	0.11
P01876	Immunoglobulin heavy constant alpha 1	*IGHA1*	0.001	-0.02	0.03	0.43	0.07	0.00	0.00	0.06	0.09	0.00	0.01	0.05	0.06	0.01	0.01	0.02	0.04	0.00	0.09
P01834	Immunoglobulin kappa constant	*IGKC*	0.000	-0.01	0.01	0.13	0.02	0.00	0.00	0.01	0.03	0.00	0.00	0.02	0.02	0.00	0.01	0.00	0.01	0.00	0.03
P0DOY3	Immunoglobulin lambda constant 3	*IGLC3*	0.001	-0.01	0.02	0.21	0.04	0.00	0.00	0.01	0.05	0.00	0.01	0.03	0.03	0.01	0.01	0.01	0.02	0.00	0.04
P06870	Kallikrein-1	*KLK1*	0.025	-0.31	0.53	7.28	1.26	0.00	0.02	0.72	1.02	0.00	0.22	0.72	1.09	0.13	0.32	0.40	0.61	0.23	1.25
P01042	Kininogen-1	*KNG1*	0.009	-0.10	0.18	2.50	0.43	0.00	0.02	0.61	0.35	-0.01	0.06	0.29	0.30	0.10	0.11	0.13	0.10	0.01	0.62
Q16363	Laminin subunit alpha-4	*LAMA4*	0.004	-0.05	0.08	1.15	0.20	0.00	0.04	0.79	0.16	0.00	0.03	0.11	0.16	0.04	0.05	0.15	0.06	0.01	0.17
Q14847	LIM and SH3 domain protein 1	*LASP1*	0.001	-0.02	0.03	0.44	0.08	0.00	0.00	0.04	0.09	0.00	0.01	0.04	0.05	0.02	0.02	0.02	0.01	0.00	0.11
P98164	Low-density lipoprotein receptor-related protein 2	*LRP2*	0.006	-0.08	0.14	1.85	0.32	0.01	0.15	3.30	0.24	0.00	0.06	0.21	0.22	0.06	0.09	0.03	0.16	0.03	0.42
P61626	Lysozyme C	*LYZ*	0.000	0.00	0.01	0.08	0.01	0.00	0.00	0.00	0.01	0.00	0.00	0.01	0.01	0.00	0.00	0.01	0.00	0.00	0.01
P09603	Macrophage colony-stimulating factor 1	*CSF1*	0.002	-0.02	0.04	0.56	0.10	0.00	0.00	0.12	0.14	0.00	0.01	0.06	0.08	0.02	0.02	0.04	0.01	0.00	0.14
P08582	Melanotransferrin	*MELTF*	0.000	0.00	0.00	0.06	0.01	0.00	0.00	0.02	0.01	0.00	0.00	0.00	0.01	0.00	0.00	0.01	0.00	0.00	0.01

Q13477	Mucosal addressin cell adhesion molecule 1	*MADCAM1*	0.002	-0.03	0.05	0.66	0.11	0.00	0.01	0.09	0.14	0.00	0.01	0.06	0.12	0.02	0.02	0.02	0.04	0.00	0.17
P14543	Nidogen-1	*NID1*	0.002	-0.02	0.04	0.56	0.10	0.00	0.01	0.26	0.09	0.00	0.02	0.06	0.08	0.02	0.02	0.01	0.03	0.01	0.15
P10451	Osteopontin	*SPP1*	0.030	-0.37	0.64	8.71	1.50	0.00	0.00	1.05	1.59	-0.03	0.17	0.97	0.85	0.27	0.71	0.78	0.21	0.00	1.99
P0DJD9	Pepsin A-5	*PGA5*	0.004	-0.04	0.08	1.05	0.18	0.00	0.00	0.15	0.16	0.00	0.04	0.11	0.16	0.02	0.04	0.06	0.11	0.02	0.17
Q6UXB8	Peptidase inhibitor 16	*PI16*	0.011	-0.14	0.24	3.23	0.56	0.00	0.03	0.54	0.58	0.00	0.05	0.30	0.53	0.08	0.13	0.14	0.06	0.00	0.89
Q96FE7	Phosphoinositide-3-kinase-interacting protein 1	*PIK3IP1*	0.006	-0.07	0.13	1.74	0.30	0.00	0.00	0.17	0.20	0.00	0.04	0.15	0.29	0.05	0.07	0.13	0.06	0.02	0.39
P05155	Plasma protease C1 inhibitor	*SERPING1*	0.003	-0.03	0.06	0.75	0.13	0.00	0.01	0.14	0.09	0.00	0.02	0.09	0.11	0.02	0.02	0.09	0.03	0.01	0.12
P01133	Pro-epidermal growth factor	*EGF*	0.010	-0.12	0.21	2.82	0.49	0.03	0.04	1.29	0.47	0.00	0.07	0.28	0.40	0.09	0.12	0.03	0.21	0.03	0.70
P28799	Progranulin	*GRN*	0.002	-0.02	0.04	0.50	0.09	0.00	0.01	0.11	0.09	0.00	0.01	0.07	0.07	0.01	0.02	0.00	0.00	0.00	0.17
P07602	Prosaposin	*PSAP*	0.006	-0.08	0.13	1.80	0.31	0.00	0.01	0.36	0.29	0.00	0.03	0.16	0.27	0.06	0.08	0.36	0.00	0.01	0.23
P02760	Protein AMBP	*AMBP*	0.642	-7.90	13.77	188.14	32.49	0.00	3.21	25.06	31.54	-0.19	6.21	16.79	27.20	6.75	7.30	12.05	9.86	1.64	40.70
P00734	Prothrombin	*F2*	0.002	-0.03	0.05	0.66	0.11	0.00	0.01	0.16	0.09	0.00	0.02	0.06	0.09	0.03	0.03	0.03	0.02	0.01	0.16
P18827	Syndecan-1	*SDC1*	0.001	-0.01	0.02	0.22	0.04	0.00	0.00	0.02	0.04	0.00	0.00	0.02	0.04	0.00	0.01	0.01	0.00	0.00	0.07
P31431	Syndecan-4	*SDC4*	0.008	-0.10	0.18	2.44	0.42	0.00	0.00	0.18	0.42	0.00	0.06	0.13	0.41	0.08	0.15	0.17	0.03	0.03	0.61

	P07204	Thrombomodulin	*THBD*	0.000	0.00	0.00	0.06	0.01	0.00	0.00	0.01	0.01	0.00	0.00	0.01	0.01	0.00	0.00	0.00	0.00	0.00	0.01
	P02766	Transthyretin	*TTR*	0.001	-0.01	0.01	0.17	0.03	0.00	0.00	0.01	0.02	0.00	0.01	0.01	0.03	0.01	0.01	0.01	0.02	0.00	0.03
	P08138	Tumor necrosis factor receptor superfamily member 16	*NGFR*	0.001	-0.02	0.03	0.44	0.08	0.00	0.00	0.07	0.09	0.00	0.01	0.05	0.07	0.01	0.02	0.03	0.01	0.00	0.11
	P30530	Tyrosine-protein kinase receptor UFO	*AXL*	0.003	-0.04	0.07	1.00	0.17	0.00	0.01	0.33	0.18	0.00	0.03	0.09	0.16	0.03	0.04	0.06	0.06	0.01	0.22
	P07911	Uromodulin	*UMOD*	0.100	-1.23	2.14	29.17	5.04	0.20	0.30	6.95	4.04	-0.01	0.86	3.33	3.98	0.72	1.07	0.05	1.79	0.06	8.06
	Q6EMK4	Vasorin	*VASN*	0.001	-0.01	0.02	0.24	0.04	0.00	0.01	0.06	0.05	0.00	0.00	0.02	0.05	0.01	0.01	0.02	0.01	0.00	0.05
	Q12907	Vesicular integral-membrane protein VIP36	*LMAN2*	0.000	0.00	0.00	0.06	0.01	0.00	0.00	0.01	0.01	0.00	0.00	0.00	0.01	0.00	0.00	0.00	0.00	0.00	0.01
	P22891	Vitamin K-dependent protein Z	*PROZ*	0.001	-0.01	0.02	0.31	0.05	0.00	0.00	0.05	0.04	0.00	0.01	0.03	0.04	0.01	0.01	0.03	0.02	0.01	0.05
	P04004	Vitronectin	*VTN*	0.010	-0.12	0.21	2.80	0.48	0.00	0.02	0.52	0.46	-0.01	0.11	0.23	0.37	0.11	0.13	0.07	0.12	0.05	0.71
SFQ6	P63261	Actin, cytoplasmic 2	*ACTG1*	0.016	-0.17	0.52	4.10	0.79	0.00	0.03	0.66	0.60	0.00	0.13	0.37	0.71	0.16	0.21	0.58	0.33	0.10	0.57
	O00468	Agrin	*AGRN*	0.001	-0.01	0.04	0.30	0.06	0.00	0.02	0.25	0.06	0.00	0.01	0.03	0.06	0.01	0.01	0.01	0.02	0.00	0.09
	P01011	Alpha-1-antichymotrypsin	*SERPINA3*	0.004	-0.04	0.12	0.92	0.18	0.00	0.01	0.17	0.15	0.00	0.03	0.09	0.15	0.03	0.04	0.15	0.06	0.01	0.13
	P01009	Alpha-1-antitrypsin	*SERPINA1*	0.008	-0.09	0.28	2.19	0.42	0.00	0.02	0.39	0.27	0.00	0.07	0.22	0.36	0.08	0.11	0.36	0.13	0.04	0.31
	P08519	Apolipoprotein(a)	*LPA*	0.004	-0.04	0.14	1.07	0.21	0.00	0.00	0.93	0.19	0.00	0.04	0.15	0.15	0.03	0.04	0.02	0.05	0.02	0.32

P98160	Basement membrane-specific heparan sulfate proteoglycan core protein	*HSPG2*	0.002	-0.02	0.07	0.54	0.11	0.00	0.04	0.98	0.10	0.00	0.01	0.07	0.10	0.02	0.02	0.01	0.06	0.01	0.13
P16278	Beta-galactosidase	*GLB1*	0.001	-0.01	0.04	0.31	0.06	0.00	0.00	0.09	0.05	0.00	0.01	0.03	0.05	0.01	0.01	0.02	0.03	0.01	0.06
P21810	Biglycan	*BGN*	0.001	-0.01	0.02	0.14	0.03	0.00	0.00	0.02	0.02	0.00	0.00	0.01	0.02	0.01	0.01	0.02	0.01	0.00	0.02
P19835	Bile salt-activated lipase	*CEL*	0.003	-0.03	0.11	0.84	0.16	0.00	0.01	0.25	0.17	0.00	0.03	0.07	0.17	0.02	0.03	0.08	0.04	0.01	0.20
P22792	Carboxypeptidase N subunit 2	*CPN2*	0.002	-0.02	0.08	0.59	0.11	0.00	0.01	0.14	0.10	0.00	0.02	0.07	0.10	0.01	0.02	0.10	0.02	0.01	0.09
P16070	CD44 antigen	*CD44*	0.094	-0.98	3.10	24.39	4.72	0.00	0.28	7.64	3.72	-0.07	0.66	3.65	3.17	0.68	1.21	0.75	0.10	0.03	8.50
Q9BY67	Cell adhesion molecule 1	*CADM1*	0.004	-0.04	0.12	0.95	0.18	0.00	0.01	0.18	0.12	0.00	0.03	0.10	0.16	0.03	0.04	0.04	0.10	0.02	0.20
P10909	Clusterin	*CLU*	0.035	-0.37	1.17	9.20	1.78	0.00	0.00	1.86	1.87	-0.02	0.31	1.05	1.20	0.46	0.53	2.32	0.24	0.03	0.95
P01024	Complement C3	*C3*	0.000	-0.01	0.02	0.13	0.02	0.00	0.00	0.09	0.02	0.00	0.00	0.01	0.02	0.01	0.01	0.01	0.01	0.00	0.02
O60494	Cubilin	*CUBN*	0.002	-0.02	0.07	0.53	0.10	0.01	0.01	0.81	0.09	0.00	0.02	0.07	0.08	0.01	0.02	0.01	0.06	0.01	0.13
P24855	Deoxyribonuclease-1	*DNASE1*	0.002	-0.02	0.06	0.44	0.09	0.00	0.00	0.05	0.06	0.00	0.02	0.04	0.08	0.01	0.02	0.05	0.04	0.00	0.07
Q9HCU0	Endosialin	*CD248*	0.006	-0.06	0.19	1.53	0.30	0.01	0.02	0.48	0.35	0.00	0.04	0.17	0.29	0.04	0.06	0.03	0.07	0.01	0.48
P35555	Fibrillin-1	*FBN1*	0.002	-0.02	0.06	0.49	0.09	0.08	0.01	0.59	0.08	0.00	0.01	0.07	0.07	0.02	0.02	0.00	0.01	0.01	0.17
P02671	Fibrinogen alpha chain	*FGA*	0.017	-0.18	0.58	4.55	0.88	0.02	0.10	1.66	0.71	-0.01	0.15	0.55	0.63	0.20	0.23	0.40	0.12	0.06	1.16

P02751	Fibronectin	*FN1*	0.004	-0.05	0.15	1.15	0.22	0.00	0.03	1.20	0.18	0.00	0.04	0.15	0.18	0.04	0.05	0.01	0.13	0.01	0.30
Q08380	Galectin-3-binding protein	*LGALS3BP*	0.221	-2.31	7.31	57.46	11.11	0.00	0.44	14.43	8.59	-0.03	2.57	6.08	9.21	1.81	2.42	4.42	4.00	0.72	12.95
Q8NBJ4	Golgi membrane protein 1	*GOLM1*	0.013	-0.14	0.43	3.39	0.66	0.00	0.07	0.59	0.71	-0.01	0.04	0.36	0.50	0.17	0.23	0.83	0.01	0.00	0.45
Q8WWV6	High affinity immunoglobulin alpha and immunoglobulin mu Fc receptor	*FCAMR*	0.001	-0.01	0.03	0.23	0.04	0.00	0.00	0.05	0.05	0.00	0.00	0.02	0.04	0.01	0.01	0.00	0.01	0.00	0.07
P01876	Immunoglobulin heavy constant alpha 1	*IGHA1*	0.007	-0.07	0.24	1.85	0.36	0.00	0.01	0.30	0.42	0.00	0.05	0.24	0.31	0.05	0.06	0.08	0.17	0.01	0.44
P01591	Immunoglobulin J chain	*JCHAIN*	0.001	-0.01	0.03	0.22	0.04	0.00	0.00	0.02	0.04	0.00	0.01	0.02	0.03	0.01	0.01	0.02	0.03	0.00	0.04
P06870	Kallikrein-1	*KLK1*	0.060	-0.63	1.99	15.61	3.02	0.00	0.06	1.73	2.47	-0.01	0.53	1.74	2.64	0.32	0.78	0.96	1.47	0.55	3.02
P01042	Kininogen-1	*KNG1*	0.035	-0.36	1.14	8.98	1.74	0.00	0.07	2.49	1.42	-0.03	0.26	1.17	1.20	0.39	0.44	0.51	0.40	0.02	2.52
Q16363	Laminin subunit alpha-4	*LAMA4*	0.006	-0.06	0.20	1.59	0.31	0.00	0.06	1.24	0.25	0.00	0.05	0.18	0.24	0.07	0.08	0.23	0.09	0.02	0.27
P98164	Low-density lipoprotein receptor-related protein 2	*LRP2*	0.081	-0.85	2.69	21.12	4.08	0.08	1.87	42.38	3.04	-0.04	0.74	2.65	2.87	0.71	1.14	0.33	2.01	0.34	5.44
O43451	Maltase-glucoamylase	*MGAM*	0.001	-0.01	0.03	0.23	0.04	0.00	0.01	0.27	0.03	0.00	0.01	0.02	0.04	0.01	0.01	0.02	0.02	0.00	0.05
O00187	Mannan-binding lectin serine protease 2	*MASP2*	0.002	-0.02	0.06	0.44	0.08	0.00	0.00	0.13	0.07	0.00	0.02	0.05	0.07	0.01	0.02	0.01	0.04	0.01	0.11
Q96DR8	Mucin-like protein 1	*MUCL1*	0.001	-0.01	0.02	0.17	0.03	0.00	0.00	0.01	0.02	0.00	0.00	0.02	0.04	0.00	0.01	0.02	0.01	0.00	0.04
Q13477	Mucosal addressin cell adhesion molecule 1	*MADCAM1*	0.002	-0.02	0.06	0.49	0.10	0.00	0.01	0.08	0.12	0.00	0.01	0.05	0.10	0.01	0.02	0.01	0.03	0.00	0.14

P10451	Osteopontin	*SPP1*	0.028	-0.30	0.94	7.39	1.43	0.00	0.00	1.01	1.52	-0.03	0.16	0.92	0.81	0.26	0.68	0.74	0.20	0.00	1.90
Q9BXP8	Pappalysin-2	*PAPPA2*	0.000	-0.01	0.02	0.13	0.02	0.00	0.00	0.10	0.03	0.00	0.00	0.01	0.02	0.00	0.01	0.01	0.01	0.00	0.03
P0DJD9	Pepsin A-5	*PGA5*	0.026	-0.28	0.87	6.85	1.32	0.00	0.00	1.11	1.18	0.00	0.29	0.79	1.18	0.12	0.26	0.44	0.79	0.14	1.27
Q6UXB8	Peptidase inhibitor 16	*PI16*	0.037	-0.39	1.23	9.69	1.87	0.00	0.11	1.84	1.97	-0.01	0.19	1.03	1.79	0.28	0.44	0.47	0.21	0.02	3.03
Q96FE7	Phosphoinositide-3-kinase-interacting protein 1	*PIK3IP1*	0.012	-0.12	0.39	3.08	0.60	0.00	0.00	0.33	0.40	0.00	0.07	0.30	0.58	0.09	0.14	0.26	0.11	0.04	0.77
P05155	Plasma protease C1 inhibitor	*SERPING1*	0.008	-0.09	0.28	2.17	0.42	0.00	0.02	0.46	0.28	0.00	0.06	0.28	0.35	0.07	0.08	0.30	0.11	0.03	0.39
Q9HCN6	Platelet glycoprotein VI	*GP6*	0.001	-0.01	0.03	0.23	0.05	0.00	0.00	0.03	0.05	0.00	0.01	0.02	0.04	0.01	0.01	0.01	0.02	0.00	0.06
O00592	Podocalyxin	*PODXL*	0.002	-0.02	0.05	0.42	0.08	0.00	0.00	0.09	0.08	0.00	0.00	0.06	0.06	0.01	0.02	0.02	0.01	0.00	0.13
P01133	Pro-epidermal growth factor	*EGF*	0.063	-0.66	2.08	16.33	3.16	0.19	0.25	8.41	3.06	-0.02	0.48	1.84	2.60	0.59	0.77	0.18	1.34	0.18	4.58
P28799	Progranulin	*GRN*	0.008	-0.08	0.25	1.96	0.38	0.00	0.02	0.48	0.39	0.00	0.04	0.29	0.29	0.06	0.07	0.01	0.00	0.00	0.75
P07602	Prosaposin	*PSAP*	0.009	-0.10	0.31	2.41	0.47	0.00	0.02	0.54	0.44	0.00	0.05	0.25	0.41	0.09	0.13	0.55	0.01	0.02	0.35
P02760	Protein AMBP	*AMBP*	1.475	-15.45	48.84	383.62	74.20	0.00	7.37	57.52	72.40	-0.44	14.25	38.55	62.43	15.50	16.76	27.65	22.62	3.78	93.43
Q7Z3E1	Protein mono-ADP-ribosyltransferase TIPARP	*TIPARP*	0.001	-0.01	0.03	0.23	0.05	0.00	0.00	0.07	0.05	0.00	0.01	0.03	0.03	0.01	0.01	0.02	0.01	0.00	0.06
P00734	Prothrombin	*F2*	0.003	-0.03	0.09	0.74	0.14	0.00	0.01	0.20	0.12	0.00	0.03	0.08	0.11	0.03	0.04	0.04	0.03	0.01	0.20

	P31431	Syndecan-4	*SDC4*	0.054	-0.57	1.79	14.10	2.73	0.00	0.00	1.17	2.71	-0.01	0.38	0.88	2.68	0.49	0.99	1.12	0.16	0.16	3.97
	P02766	Transthyretin	*TTR*	0.001	-0.01	0.03	0.24	0.05	0.00	0.00	0.01	0.03	0.00	0.01	0.02	0.04	0.01	0.01	0.01	0.03	0.00	0.05
	P08138	Tumor necrosis factor receptor superfamily member 16	*NGFR*	0.006	-0.06	0.18	1.44	0.28	0.00	0.01	0.25	0.33	0.00	0.02	0.18	0.24	0.04	0.07	0.10	0.04	0.01	0.40
	P30530	Tyrosine-protein kinase receptor UFO	*AXL*	0.004	-0.04	0.14	1.07	0.21	0.00	0.01	0.40	0.22	0.00	0.03	0.10	0.20	0.03	0.05	0.07	0.07	0.01	0.26
	P07911	Uromodulin	*UMOD*	0.269	-2.82	8.92	70.08	13.55	0.54	0.81	18.80	10.92	-0.03	2.32	9.01	10.78	1.94	2.90	0.13	4.84	0.17	21.81
	Q6EMK4	Vasorin	*VASN*	0.007	-0.08	0.25	1.94	0.38	0.00	0.06	0.54	0.41	0.00	0.03	0.19	0.40	0.06	0.06	0.22	0.08	0.03	0.42
	P22891	Vitamin K-dependent protein Z	*PROZ*	0.002	-0.02	0.07	0.56	0.11	0.00	0.01	0.10	0.09	0.00	0.02	0.06	0.09	0.02	0.03	0.05	0.04	0.02	0.10
	P04004	Vitronectin	*VTN*	0.016	-0.17	0.53	4.15	0.80	0.00	0.03	0.87	0.77	-0.01	0.19	0.39	0.61	0.19	0.22	0.11	0.20	0.09	1.19
SFQ7	Q8TB40	(Lyso)-N-acylphosphatidylethanolamine lipase	*ABHD4*	0.005	0.00	0.17	1.48	0.28	0.00	0.01	0.20	0.24	0.00	0.05	0.12	0.23	0.06	0.06	0.23	0.07	0.02	0.20
	Q9BXX2	Ankyrin repeat domain-containing protein 30B	*ANKRD30 B*	0.015	0.01	0.47	4.17	0.79	0.00	0.06	2.30	0.65	-0.01	0.08	0.43	0.50	0.22	0.24	0.75	0.07	0.08	0.55
	Q6PI47	BTB/POZ domain-containing protein KCTD18	*KCTD18*	0.001	0.00	0.02	0.19	0.04	0.00	0.00	0.03	0.03	0.00	0.00	0.02	0.03	0.01	0.01	0.02	0.01	0.00	0.04
	P16070	CD44 antigen	*CD44*	0.169	0.10	5.40	48.20	9.19	0.00	0.51	13.74	6.69	-0.13	1.18	6.56	5.70	1.23	2.18	1.34	0.18	0.05	15.28
	Q96A65	Exocyst complex component 4	*EXOC4*	0.001	0.00	0.02	0.19	0.04	0.00	0.00	0.07	0.03	0.00	0.00	0.02	0.03	0.01	0.01	0.05	0.00	0.00	0.01
	Q08380	Galectin-3-binding protein	*LGALS3BP*	0.139	0.08	4.46	39.79	7.58	0.00	0.28	9.09	5.41	-0.02	1.62	3.83	5.80	1.14	1.52	2.78	2.52	0.45	8.16

P01591	Immunoglobulin J chain	*JCHAIN*	0.003	0.00	0.09	0.84	0.16	0.00	0.00	0.05	0.14	0.00	0.02	0.08	0.11	0.04	0.05	0.06	0.09	0.01	0.13
P0DOY3	Immunoglobulin lambda constant 3	*IGLC3*	0.005	0.00	0.18	1.57	0.30	0.00	0.00	0.06	0.40	0.00	0.04	0.21	0.21	0.05	0.05	0.05	0.16	0.04	0.31
P06213	Insulin receptor	*INSR*	0.015	0.01	0.47	4.17	0.79	0.00	0.12	2.28	0.70	-0.01	0.14	0.39	0.59	0.16	0.19	0.32	0.26	0.04	0.84
P06870	Kallikrein-1	*KLK1*	0.056	0.03	1.81	16.14	3.08	0.00	0.06	1.63	2.32	-0.01	0.50	1.64	2.48	0.30	0.73	0.90	1.38	0.52	2.84
Q38SD2	Leucine-rich repeat serine/threonine-protein kinase 1	*LRRK1*	0.001	0.00	0.02	0.19	0.04	0.00	0.01	0.15	0.03	0.00	0.01	0.02	0.03	0.01	0.01	0.03	0.01	0.00	0.03
P98164	Low-density lipoprotein receptor-related protein 2	*LRP2*	0.006	0.00	0.18	1.62	0.31	0.01	0.13	2.96	0.21	0.00	0.05	0.19	0.20	0.05	0.08	0.02	0.14	0.02	0.38
P30414	NK-tumor recognition protein	*NKTR*	0.004	0.00	0.13	1.19	0.23	0.00	0.01	0.69	0.36	-0.01	0.02	0.15	0.10	0.09	0.05	0.04	0.02	0.01	0.35
P0DJD9	Pepsin A-5	*PGA5*	0.012	0.01	0.39	3.51	0.67	0.00	0.00	0.52	0.55	0.00	0.13	0.37	0.55	0.05	0.12	0.21	0.37	0.06	0.59
Q6UXB8	Peptidase inhibitor 16	*PI16*	0.005	0.00	0.16	1.44	0.28	0.00	0.02	0.25	0.27	0.00	0.03	0.14	0.24	0.04	0.06	0.06	0.03	0.00	0.41
Q96FE7	Phosphoinositide-3-kinase-interacting protein 1	*PIK3IP1*	0.007	0.00	0.24	2.13	0.41	0.00	0.00	0.21	0.25	0.00	0.05	0.19	0.37	0.06	0.09	0.16	0.07	0.03	0.48
Q8N9I9	Probable E3 ubiquitin-protein ligase DTX3	*DTX3*	0.001	0.00	0.02	0.19	0.04	0.00	0.00	0.03	0.03	0.00	0.00	0.02	0.03	0.01	0.01	0.02	0.01	0.00	0.04
P01133	Pro-epidermal growth factor	*EGF*	0.028	0.02	0.91	8.09	1.54	0.08	0.11	3.79	1.38	-0.01	0.22	0.83	1.17	0.26	0.35	0.08	0.60	0.08	2.06
P02760	Protein AMBP	*AMBP*	0.402	0.24	12.90	115.10	21.93	0.00	2.01	15.69	19.75	-0.12	3.89	10.52	17.03	4.23	4.57	7.54	6.17	1.03	25.49
Q96JQ0	Protocadherin-16	*DCHS1*	0.020	0.01	0.65	5.81	1.11	0.55	0.33	7.04	0.85	0.00	0.11	0.48	1.06	0.14	0.24	0.09	0.70	0.06	1.18

	Q9BYP7	Serine/threonine-protein kinase WNK3	*WNK3*	0.039	0.02	1.24	11.04	2.10	0.00	0.23	7.65	2.19	-0.02	0.22	1.28	1.45	0.42	0.48	0.51	0.12	0.05	3.17
	Q8N614	Transmembrane protein 156	*TMEM156*	0.004	0.00	0.12	1.04	0.20	0.00	0.01	0.12	0.14	0.00	0.03	0.12	0.14	0.04	0.04	0.10	0.07	0.00	0.19
	P07911	Uromodulin	*UMOD*	0.053	0.03	1.71	15.23	2.90	0.11	0.16	3.71	2.16	-0.01	0.46	1.78	2.13	0.38	0.57	0.03	0.96	0.03	4.31
	Q14508	WAP four-disulfide core domain protein 2	*WFDC2*	0.003	0.00	0.09	0.78	0.15	0.00	0.00	0.04	0.12	0.00	0.01	0.11	0.11	0.02	0.02	0.04	0.00	0.01	0.22
	Q702N8	Xin actin-binding repeat-containing protein 1	*XIRP1*	0.007	0.00	0.22	1.93	0.37	0.00	0.08	1.34	0.42	0.00	0.02	0.19	0.31	0.07	0.08	0.14	0.04	0.01	0.49
SFQ8	Q12797	Aspartyl/asparaginyl beta-hydroxylase	*ASPH*	0.046	0.83	0.73	11.53	2.13	0.05	0.09	3.94	2.34	-0.04	0.34	1.07	1.76	0.56	0.85	1.96	0.30	0.11	2.22
	Q9UHQ4	B-cell receptor-associated protein 29	*BCAP29*	0.001	0.01	0.01	0.17	0.03	0.00	0.00	0.02	0.03	0.00	0.01	0.02	0.02	0.01	0.01	0.06	0.00	0.00	0.00
	P49747	Cartilage oligomeric matrix protein	*COMP*	0.006	0.11	0.10	1.58	0.29	0.01	0.03	0.52	0.27	0.00	0.04	0.19	0.24	0.06	0.11	0.03	0.04	0.01	0.54
	P16070	CD44 antigen	*CD44*	0.172	3.13	2.73	43.22	7.97	0.00	0.52	14.02	6.82	-0.13	1.21	6.70	5.82	1.25	2.23	1.37	0.19	0.05	15.60
	Q5TB80	Centrosomal protein of 162 kDa	*CEP162*	0.000	0.01	0.01	0.11	0.02	0.00	0.00	0.07	0.02	0.00	0.00	0.01	0.01	0.01	0.01	0.04	0.00	0.00	0.00
	Q6ZUS5	Coiled-coil domain-containing protein 121	*CCDC121*	0.001	0.01	0.01	0.20	0.04	0.00	0.00	0.03	0.06	0.00	0.00	0.02	0.02	0.02	0.01	0.07	0.00	0.00	0.01
	O14802	DNA-directed RNA polymerase III subunit RPC1	*POLR3A*	0.006	0.11	0.10	1.51	0.28	0.00	0.05	0.93	0.22	0.00	0.04	0.14	0.26	0.08	0.07	0.26	0.08	0.03	0.22
	Q5XPI4	E3 ubiquitin-protein ligase RNF123	*RNF123*	0.004	0.07	0.06	0.98	0.18	0.00	0.05	0.58	0.19	0.00	0.03	0.10	0.17	0.04	0.05	0.18	0.04	0.01	0.16
	Q86UK7	E3 ubiquitin-protein ligase ZNF598	*ZNF598*	0.000	0.01	0.01	0.11	0.02	0.00	0.00	0.04	0.03	0.00	0.00	0.01	0.02	0.01	0.00	0.01	0.00	0.00	0.03

Q5CZC0	Fibrous sheath-interacting protein 2	*FSIP2*	0.000	0.01	0.01	0.11	0.02	0.00	0.01	0.33	0.02	0.00	0.00	0.01	0.02	0.01	0.01	0.02	0.00	0.00	0.02
Q08380	Galectin-3-binding protein	*LGALS3BP*	0.281	5.10	4.45	70.53	13.01	0.00	0.56	18.34	10.92	-0.04	3.26	7.72	11.71	2.30	3.07	5.61	5.09	0.91	16.46
Q8N1C3	Gamma-aminobutyric acid receptor subunit gamma-1	*GABRG1*	0.007	0.13	0.11	1.75	0.32	0.00	0.01	0.37	0.21	0.00	0.09	0.18	0.28	0.07	0.07	0.22	0.16	0.02	0.30
Q7Z4P5	Growth/differentiation factor 7	*GDF7*	0.007	0.13	0.11	1.78	0.33	0.00	0.04	0.33	0.42	0.00	0.04	0.14	0.39	0.09	0.06	0.16	0.08	0.01	0.46
Q6WRI0	Immunoglobulin superfamily member 10	*IGSF10*	0.000	0.01	0.01	0.11	0.02	0.00	0.00	0.12	0.02	0.00	0.00	0.01	0.02	0.00	0.00	0.00	0.01	0.00	0.03
P06870	Kallikrein-1	*KLK1*	0.054	0.99	0.86	13.63	2.51	0.00	0.05	1.57	2.23	-0.01	0.48	1.57	2.38	0.29	0.70	0.87	1.33	0.50	2.73
A4D0S4	Laminin subunit beta-4	*LAMB4*	0.001	0.02	0.01	0.23	0.04	0.00	0.01	0.18	0.04	0.00	0.01	0.03	0.04	0.01	0.01	0.03	0.01	0.00	0.05
P48449	Lanosterol synthase	*LSS*	0.000	0.01	0.01	0.11	0.02	0.00	0.00	0.04	0.02	0.00	0.00	0.01	0.02	0.00	0.00	0.02	0.00	0.00	0.02
P62913	Large ribosomal subunit protein uL5	*RPL11*	0.007	0.13	0.11	1.80	0.33	0.00	0.00	0.15	0.27	0.00	0.06	0.14	0.30	0.13	0.09	0.23	0.14	0.03	0.31
Q9H7P6	Multivesicular body subunit 12B	*MVB12B*	0.009	0.17	0.15	2.35	0.43	0.00	0.00	0.33	0.54	0.00	0.07	0.26	0.39	0.11	0.10	0.14	0.16	0.01	0.63
Q9Y239	Nucleotide-binding oligomerization domain-containing protein 1	*NOD1*	0.005	0.09	0.08	1.28	0.24	0.00	0.07	0.55	0.22	0.00	0.05	0.14	0.22	0.05	0.06	0.28	0.05	0.02	0.16
Q86WC4	Osteopetrosis-associated transmembrane protein 1	*OSTM1*	0.006	0.10	0.09	1.42	0.26	0.00	0.01	0.21	0.32	0.00	0.04	0.19	0.23	0.05	0.06	0.27	0.05	0.01	0.24
Q9BXB5	Oxysterol-binding protein-related protein 10	*OSBPL10*	0.001	0.01	0.01	0.20	0.04	0.00	0.00	0.07	0.04	0.00	0.01	0.02	0.03	0.01	0.01	0.03	0.01	0.00	0.04
Q99572	P2X purinoceptor 7	*P2RX7*	0.000	0.01	0.01	0.11	0.02	0.00	0.00	0.03	0.02	0.00	0.01	0.01	0.02	0.01	0.00	0.01	0.01	0.00	0.02

P0DJD9	Pepsin A-5	*PGA5*	0.012	0.21	0.18	2.91	0.54	0.00	0.00	0.49	0.52	0.00	0.13	0.35	0.52	0.05	0.12	0.19	0.35	0.06	0.56
Q96FE7	Phosphoinositide-3-kinase-interacting protein 1	*PIK3IP1*	0.011	0.20	0.17	2.75	0.51	0.00	0.00	0.31	0.37	0.00	0.07	0.27	0.54	0.09	0.13	0.24	0.10	0.04	0.71
Q8TCS8	Polyribonucleotide nucleotidyltransferase 1, mitochondrial	*PNPT1*	0.003	0.06	0.05	0.82	0.15	0.00	0.02	0.28	0.13	0.00	0.02	0.08	0.15	0.04	0.04	0.13	0.06	0.02	0.12
P01133	Pro-epidermal growth factor	*EGF*	0.000	0.01	0.01	0.11	0.02	0.00	0.00	0.06	0.02	0.00	0.00	0.01	0.02	0.00	0.01	0.00	0.01	0.00	0.03
P02760	Protein AMBP	*AMBP*	0.202	3.67	3.20	50.66	9.34	0.00	1.01	7.86	9.90	-0.06	1.95	5.27	8.53	2.12	2.29	3.78	3.09	0.52	12.77
Q6ZTI0	Putative uncharacterized protein FLJ44636		0.000	0.01	0.01	0.11	0.02	0.00	0.00	0.01	0.02	0.00	0.00	0.01	0.02	0.00	0.00	0.01	0.01	0.00	0.03
Q8TD19	Serine/threonine-protein kinase Nek9	*NEK9*	0.001	0.02	0.01	0.23	0.04	0.00	0.00	0.10	0.04	0.00	0.01	0.02	0.04	0.01	0.01	0.03	0.01	0.00	0.04
Q9BYP7	Serine/threonine-protein kinase WNK3	*WNK3*	0.014	0.25	0.22	3.43	0.63	0.00	0.08	2.71	0.78	-0.01	0.08	0.45	0.51	0.15	0.17	0.18	0.04	0.02	1.12
P31431	Syndecan-4	*SDC4*	0.036	0.65	0.57	8.99	1.66	0.00	0.00	0.77	1.79	0.00	0.25	0.58	1.77	0.33	0.65	0.74	0.11	0.11	2.62
Q6ZMP0	Thrombospondin type-1 domain-containing protein 4	*THSD4*	0.006	0.10	0.09	1.42	0.26	0.00	0.03	0.64	0.31	0.00	0.04	0.19	0.21	0.06	0.06	0.02	0.08	0.01	0.46
Q9NXG2	THUMP domain-containing protein 1	*THUMPD1*	0.059	1.07	0.93	14.74	2.72	0.00	0.06	2.31	2.69	-0.05	0.33	1.58	2.26	0.86	0.83	1.99	0.55	0.05	3.27
Q9NQE7	Thymus-specific serine protease	*PRSS16*	0.001	0.01	0.01	0.15	0.03	0.00	0.00	0.03	0.03	0.00	0.00	0.02	0.03	0.00	0.00	0.03	0.01	0.00	0.02
Q9NX74	tRNA-dihydrouridine(20) synthase [NAD(P)+]-like	*DUS2*	0.014	0.25	0.22	3.48	0.64	0.00	0.08	0.76	0.70	0.00	0.08	0.32	0.62	0.18	0.18	0.61	0.19	0.05	0.54
P07911	Uromodulin	*UMOD*	0.019	0.34	0.30	4.70	0.87	0.04	0.06	1.30	0.76	0.00	0.16	0.63	0.75	0.13	0.20	0.01	0.34	0.01	1.51

	Q14508	WAP four-disulfide core domain protein 2	*WFDC2*	0.008	0.15	0.13	2.02	0.37	0.00	0.00	0.10	0.36	0.00	0.03	0.31	0.34	0.05	0.07	0.12	0.01	0.04	0.64
SFQ9	P16070	CD44 antigen	*CD44*	0.127	2.90	1.87	32.28	5.82	0.00	0.38	10.34	5.03	-0.10	0.89	4.94	4.29	0.92	1.64	1.01	0.14	0.03	11.50
	Q08380	Galectin-3-binding protein	*LGALS3BP*	0.674	15.45	9.92	171.66	30.93	0.00	1.35	44.06	26.24	-0.09	7.84	18.56	28.13	5.53	7.38	13.49	12.22	2.19	39.54
	P06870	Kallikrein-1	*KLK1*	0.053	1.21	0.78	13.48	2.43	0.00	0.05	1.53	2.17	-0.01	0.46	1.54	2.32	0.28	0.69	0.85	1.29	0.48	2.67
	P02760	Protein AMBP	*AMBP*	0.134	3.07	1.98	34.17	6.16	0.00	0.67	5.24	6.59	-0.04	1.30	3.51	5.68	1.41	1.53	2.52	2.06	0.34	8.50
	Q8NFT6	Protein DBF4 homolog B	*DBF4B*	0.007	0.16	0.11	1.83	0.33	0.00	0.06	0.48	0.44	0.00	0.05	0.21	0.31	0.08	0.07	0.14	0.04	0.01	0.53
	Q0IIN9	Putative uncharacterized protein ZNF252P-AS1	*ZNF252P-AS1*	0.004	0.10	0.06	1.11	0.20	0.00	0.02	0.10	0.32	0.00	0.01	0.12	0.21	0.06	0.05	0.07	0.06	0.04	0.26

† Relative protein abundance = Spectral intensity of each protein / Σ Spectral intensities of all proteins in the same fraction

‡ Abundance-weighted crystal-promoting activity (%) = Relative protein abundance of each protein × Crystal-promoting activity of each fraction (%)

δ Abundance-weighted physicochemical property = Relative protein abundance of each protein × Value of each physicochemical property
